# Organic Sunscreens—Is Their Placenta Permeability the Only Issue Associated with Exposure During Pregnancy? In Silico Studies of Sunscreens’ Placenta Permeability and Interactions with Selected Placental Enzymes

**DOI:** 10.3390/molecules29245836

**Published:** 2024-12-11

**Authors:** Anna W. Sobańska, Andrzej M. Sobański

**Affiliations:** 1 Department of Analytical Chemistry, Medical University of Lodz, Muszyńskiego 1, 90-151 Lodz, Poland; 2 Faculty of Chemistry, University of Lodz, Tamka 12, 91-403 Lodz, Poland; andrzej.sobanski@edu.uni.lodz.pl

**Keywords:** organic sunscreens, placenta permeability, oxidative stress, enzyme deactivation, protein–ligand binding, molecular docking

## Abstract

One of the functions of placenta is to protect the fetus against harmful xenobiotics. Protective mechanisms of placenta are based on enzymes, e.g., antioxidant enzymes from the glutathione *S*-transferases group (GST) or human N-acetyltransferase 2 (NAT2). Many organic sunscreens are known to cross biological barriers—they are detected in mother’s milk, semen, umbilical cord blood or placental tissues. Some organic sunscreens are able to cross the placenta and to interfere with fetal development; they are known or suspected endocrine disruptors or neurotoxins. In this study, 16 organic sunscreens were investigated in the context of their placenta permeability and interactions with gluthatione S-transferase and human N-acetyltransferase 2 enzymes present in the human placenta. Binary permeability models based on discriminant analysis and artificial neural networks proved that the majority of studied compounds are likely to cross the placenta by passive diffusion. Molecular docking analysis suggested that some sunscreens show stronger affinity for glutathione S-transferase and human N-acetyltransferase 2 that native ligands (glutathione and Coenzyme A for GST and NAT2, respectively)—it is therefore possible that they are able to reduce the enzyme’s protective activity. It was established that sunscreens bind to the studied enzymes mainly by alkyl, hydrogen bonds, van der Waals, π-π, π-alkyl and π-sulfur interactions. To conclude, sunscreens may become stressors affecting humans by different mechanisms and at different stages of development.

## 1. Introduction

Organic UV filters are used in cosmetic products to protect human skin and hair against harmful effects of UV radiation and to prevent photodegradation of materials (e.g., plastics, fabrics or cosmetic products). Large quantities of these compounds circulate in the environment and affect ecosystems [1,2,3,4]. UV filters are applied on the surface of skin or hair and are expected to remain there; however, many compounds from this group are drug-like [5] and are absorbed through the skin from the gastro-intestinal or pulmonary tract [6]. Some organic sunscreens have been detected in human semen [7], mother’s milk [8], umbilical cord blood [9,10] or placental tissues [11]. UV filters are known to cause contact allergies and photoallergies [12], but there are additional health issues associated with excessive exposure to such compounds which are likely endocrine disruptors [13,14,15,16,17,18,19,20,21,22,23,24,25,26,27,28,29,30,31,32,33,34,35] and neurotoxins [36] and can interfere with the development of human and animal offspring [37,38,39,40].

Apart from physically separating maternal and fetal blood circulation and facilitating transport of compounds to and from a fetus, the placenta, with its multiple enzymatic systems, plays a crucial role in metabolism and excretion of xenobiotics [41]. Despite the significance of human placental enzymes, few studies have been conducted thus far on their inhibition by environmental pollutants; compounds investigated in this context include perfluoroalkyl and polyfluoroalkyl substances (“forever” chemicals) and nanoplastics [42,43].

The objective of this study was to assess the ability of organic sunscreens to carry out the following: (i) crossing the human placenta; and (ii) interfering with placental enzymes whose role is protecting a fetus against harmful xenobiotics. The placenta permeability of selected organic sunscreens has been quantified earlier as a part of a broader study on the pharmacokinetic properties of sunscreens and their degradation products in aquatic ecosystems [44], and the property determined in that research was ***FM*** (the equilibrium ratio of compound’s concentrations in fetal and maternal blood circulations). In the current study, the focus is on classification and binary (“High” or “Low” permeability) models based on experimental permeability data. The studied sunscreens come from all the major chemical families of UV filters (including benzophenones, salicylates, p-methoxycinnamates, triazines and PABA derivatives); their legal status and popularity are different depending on the region [45]. The enzymes whose potential deactivation by sunscreens is investigated in this study are the following: (i) glutathione s-transferases (GSTs)—due to their antioxidant properties, these enzymes are involved in the metabolism of toxins through conjugation with reduced glutathione [46]; (ii) human N-acetyltransferase 2 (NAT2), which deactivates arylamine and hydrazine compounds, many of which are harmful [41,47].

## 2. Results and Discussion

### 2.1. Human Placenta Permeability—Qualitative Studies

Earlier studies on qualitative measures of the placenta permeability of sunscreens proved that the majority of studied compounds have log ***FM*** value above the cut-off value separating molecules that are expected to cross the placenta barrier very easily from those whose placental passage is more difficult [44]. At this point, attention turned to binary classification (High/Low or PL1/PL0) models of placenta permeability based on experimental data. The statistical tools used to build classification models were general discriminant analysis (GDA) and artificial neural networks (ANNs).

Discriminant function analysis (DA) is used to select the variables which discriminate between two or more naturally occurring groups; it is a classification method, as it establishes the class membership of new cases using appropriate discriminating variables. General discriminant analysis was performed by the forward stepwise method, using 84 independent variables selected previously by the partial least squares (PLS) method (Section 3.3). The final selection of descriptors used in discriminant functions D (D_raw_—the function with raw coefficients, Equation (1) and D_std_—the function with standardized coefficients, Equation (2)) was achieved after six steps, and the variables were selected in the following order: ***Lipinski***, ***AATSC6m***, ***VSA_EState5***, ***XLOGP3***, ***ATSC2d***.

D_raw_ = 2.66 − 4.78 ***Lipinski*** − 0.096 ***ATSC2d*** + 0.33 ***XLOGP3*** + 0.33 ***VSA_EState5*** + 0.019 ***AATSC6m*** (1)

D_std_ = −1.06 ***Lipinski*** − 0.47 ***ATSC2d*** + 0.54 ***XLOGP3*** + 0.61 ***VSA_EState5*** + 0.82 ***AATSC6m*** (2)

where ***AATSC6m*** is the averaged and centered Moreau–Broto autocorrelation of lag 6 weighted by mass; ***VSA_EState5*** is the VSA EState Descriptor 5 (5.74 ≤ x < 6.00); and ***ATSC2d*** is the centered Moreau–Broto autocorrelation of lag 2 weighted by valence electrons.

The classification functions obtained by GDA for the training dataset and validated using the test set are as follows (Equations (3) and (4)):

PL0 = −13.57 − 3.60 ***Lipinski*** − 0.76 ***ATSC2d*** + 3.06 ***XLOGP3*** + 1.16 ***VSA_EState5*** + 0.050 ***AATSC6m*** (3)

PL1 = −15.77 + 30.92 ***Lipinski*** − 0.068 ***ATSC2d*** + 0.65 ***XLOGP3*** − 1.24 ***VSA_EState5*** − 0.086 ***AATSC6m*** (4)

The values of classification functions are calculated for every compound, and its class membership is determined based on the highest value of a respective classification function.

High classification potential of discriminant analysis presented at this stage of the study was reflected using the following statistical parameters: Wilk’s λ = 0.10, χ^2^ = 80.9, *p* < 0.01.

**Figure 1:**
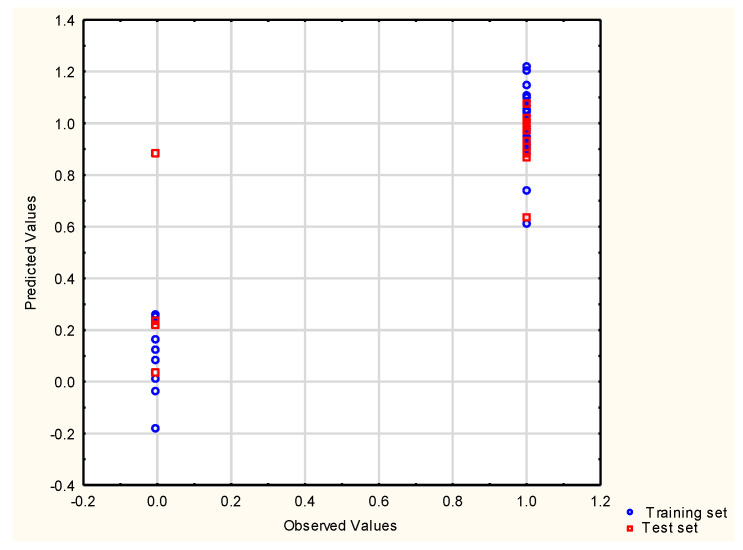
Classification by DA—probability plot.

Using DA, one compound was misclassified in the test set (Figure 1). The results of the assignment of sunscreens to PL1 or PL0 groups are presented in Table S1 (Supplementary Materials). The importance of independent variables in the DA model was determined using standardized coefficients in the discriminant function (D_std_)—the descriptor with the largest coefficient (the most important one in the prediction of class membership) is ***Lipinski***.

**Figure 2:**
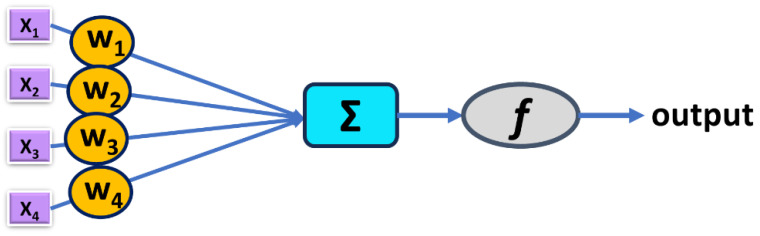
Schematic representation of an artificial neuron.

**Figure 3:**
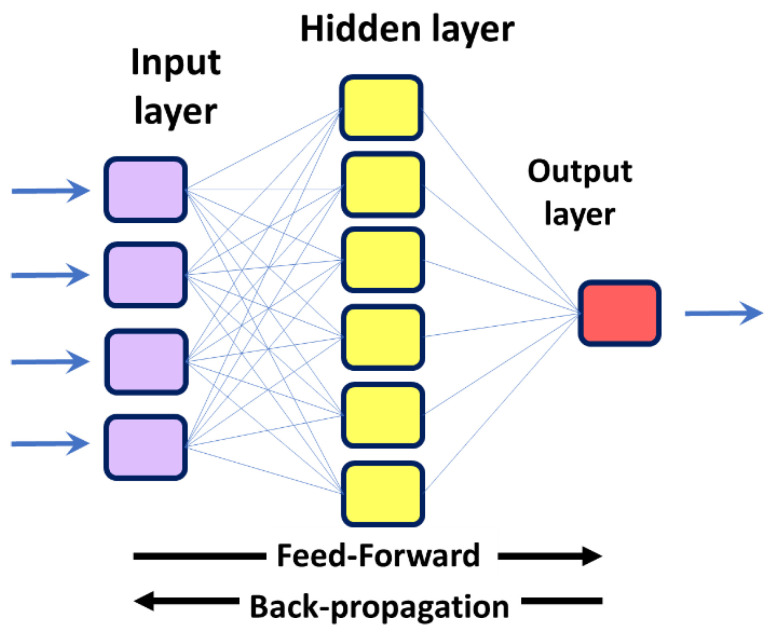
ANN architecture.

The set of five independent variables used in the GDA model was used as input data in the classification model generated using artificial neural network (ANN) methodology. ANNs are simplified, mathematical models of biological neuron networks involving neurons with different activation functions, different architectures of connections between neurons and different training methods. An artificial neuron uses weighted input data (Σ*x_i·_w_i_*) to evaluate an activation function *f* and to return the output value (Figure 2). Activation functions can be of different types—binary ({0; 1} or {−1; +1}), linear with continuous output or non-linear (e.g., hyperbolic tangent or exponential). ANNs are intended to imitate the layer topology encountered in the cerebral cortex of living organisms—in the most typical situation, the ANN involves an input layer, a hidden layer/layers and an output layer (Figure 3) [48,49]—and they aim to mimic biological neural networks’ potential for learning and creativity [50].

In the current study, all five relevant descriptors passed a global sensitivity analysis test (GSA), which evaluates every input variable in ANN models by computing sums of squared residuals for the model without that variable compared to the full model (when an input variable scores 1 or less than 1 in GSA, it means that for this particular network, this variable is redundant). We observed similar performance, with 100% accuracy in the test and the validation sets and one error (97.4% accuracy) in the training set (compound ***43***, duloxetine), for ANNs of the following architectures: (i) multilayer perceptron (MLP) network with SOS error function, hyperbolic tangent hidden activation and hyperbolic tangent output activation (six input layers, three hidden layers and two output layers); (ii) multilayer perceptron (MLP) network with SOS error function, logistic hidden activation and identity output activation (six input layers, nine hidden layers and two output layers). The results of PL0/PL1 classification of sunscreens using ANN classification models are provided in Table S1 (Supplementary Materials).

It was established that some physicochemical parameters of compounds investigated in this study (both the set of 54 reference compounds and 16 sunscreens) that are (or have been classified as) PL0 or PL1 differ significantly. Molecules that are known or expected to cross the placenta easily are smaller (***M***_w_, #***HvAt,*** #***ArHvAt***), less lipophilic (***XLOGP3***) and less polarizable (***MR***) than those whose placental passage is limited; drug-likeness according to Lipinski and Ghose is also different for the compounds in both groups (Table 1).

### 2.2. Sunscreens’ Affinity for Selected Placenta Enzymes

**Figure 4:**
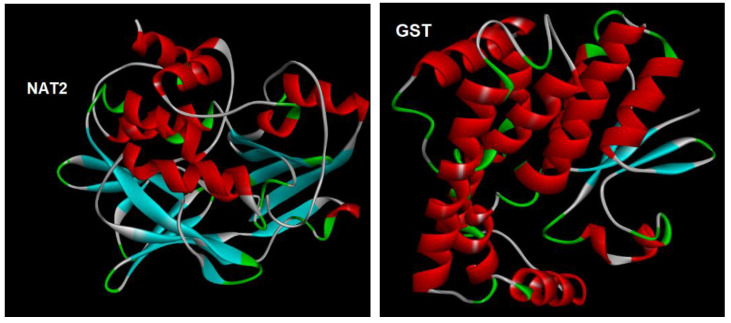
Structures of GST and NAT2 enzymes.

The binding affinities of selected organic sunscreens on GST and NAT2 enzymes (Figure 4) were calculated by molecular docking methodology as described in Section 3.4. The results are presented in Table 2 along with the values obtained for reference ligands (glutathione for GST and coenzyme A for NAT2, respectively [42]). It was established that the majority of studied sunscreens (apart from PABA and its derivatives Et-PABA and EHDP) have a potential to bind to the GST enzyme at least as strongly as glutathione. The affinity of studied compounds for the NAT2 enzyme is lower than that of the reference ligand, with the exception of BMDM, PBSA, MBC and BP-4.

Likely binding sites of both enzymes were identified (Figure S1, Supplementary Materials). Sunscreens’ binding to enzyme targets is a complex process involving large groups of aminoacids; the aminoacids that are mainly involved in different types of interactions are presented in Table 3 and Table 4. As can be seen, for the GST enzyme, the key amino acids involved in binding are leucine (Leu A35, Leu A114 and Leu 119), valine (Val A10 and Val A36), methionine (Met A236), glutamine (Gln A12) and tryptophan (Trp A115) and binding is mainly by van der Waals, alkyl, π-σ and H-bond interactions. For NAT2, the binding is mainly via phenylalanine (Phe A37, Phe A 93, Phe A217) and serine (Ser A125, Ser A129); other aminoacids involved are Gly A126, Leu A288 and Cys A68, and binding is mainly by van der Waals, π-π, alkyl and π-sulfur interactions.

**Figure 5:**
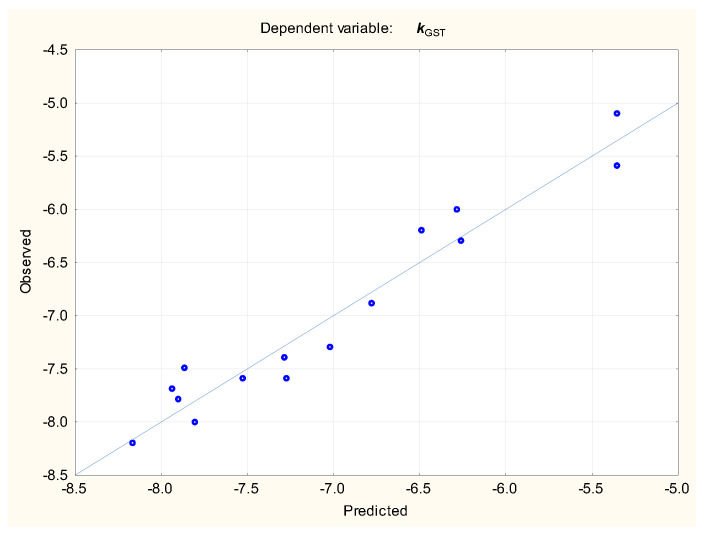
Equation (5)—***k***_GST_, predicted vs. observed values, n = 16.

**Figure 6:**
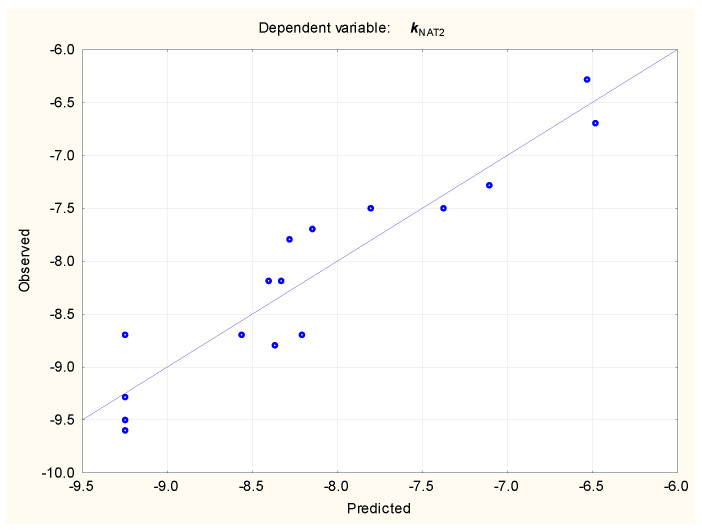
Equation (6)—***k***_NAT2_, predicted vs. observed values, n = 16.

The relationships between the binding affinities (***k***) of ligands for studied proteins (GST and NAT2) and selected electrotopological state indices [51,52] were investigated to determine if the ***k*** values for both enzymes may be described by simple, linear relationships (Equations (5) and (6), Figure 5 and Figure 6):

***k***_GST_ = −5.73 (±0.1**8**) − 0.133 (±0.020) ***SeaC2C2aa*** − 0.243 (±0.039) ***Se1C1C4s*** + 0.680 (±0.143) ***Se1C3S4ad*** + 0.238 (±0.061) ***Se1C3N1a*** (n = 16, R^2^ = 0.943, R^2^_adj._ = 0.922, F = 45.12, *p* < 0.01, s_e_ = 0.260, RMSECV = 0.335) (5)

***k***_NAT2_ = −9.24 (±0.17) + 0.750 (±0.085) ***Se1C3N1a*** + 2.02 (±0.32) ***SsssCH*** + 0.442 (±0.141) ***SsssN*** (n = 16, R^2^ = 0.880, R^2^_adj._ = 0.851, F = 29.45, *p* < 0.01, s_e_ = 0.374, RMSECV = 0.388) (6)

It would be interesting to observe that the affinities of studied sunscreens for both enzymes (***k***_NAT2_ and ***k***_GST_) are somewhat interrelated (Table 5); ***k***_GST_ is moderately correlated with the physicochemical properties of studied compounds such as size (***M***_w_, #***HvAt***), aromaticity (#***ArHvAt***) and an ability to form H-bonds (#***HA***) but not with lipophilicity (***XLOGP3***), polarity (***TPSA***), polarizability (***MR***) or flexibility (#***FBR****, **F***_Csp3_); ***k***_NAT2_ is not correlated with any physicochemical properties of molecules mentioned before and responsible, e.g., for compounds’ distribution in a living organism.

### 2.3. Clinical Relevance of Molecular Docking Ligand–Enzyme Affinity Studies

**Figure 7:**
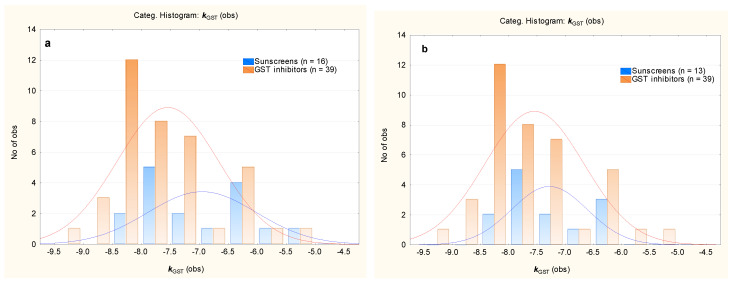
Histograms of ***k***_GST_ values for the GST inhibitors and studied sunscreens. (**a**)—16 sunscreens, (**b**)—13 sunscreens.

Enzymes from the GST family play a vital role in deactivation of xenobiotics, which is usually their great asset. However, in some therapeutic situations, similar defense mechanisms can become a disadvantage, with the activity (and, especially, the overexpression) of the GST enzymes leading to deactivation of certain drugs and to the development of drug resistance, e.g., in tumor cells [46]. With this in mind, several GST inhibitors have been developed or an inhibitory function of already known drugs has been discovered and, in the most promising cases, assessed in vitro [53,54,55,56,57,58,59]. The GST enzymes’ inhibitors come from different chemical families, and a large group of such compounds was investigated in this study, in the context of these compounds’ affinity for the human placental GST enzyme by molecular docking, to determine if their ***k***_GST_ values (provided in Supplementary Materials) were within a range that was similar to that of the studied sunscreens, with mean ***k***_GST_ = −7.0 and −7.6 for sunscreens and GST inhibitors, respectively (Figure 7a). A total of 13 sunscreens of particular concern (with an affinity for the GST enzyme that is stronger than that of glutathione) fit the range observed for the control group even better, with mean ***k***_GST_ = −7.3 and −7.6 for sunscreens and GST inhibitors, respectively (Figure 7b).

**Figure 8:**
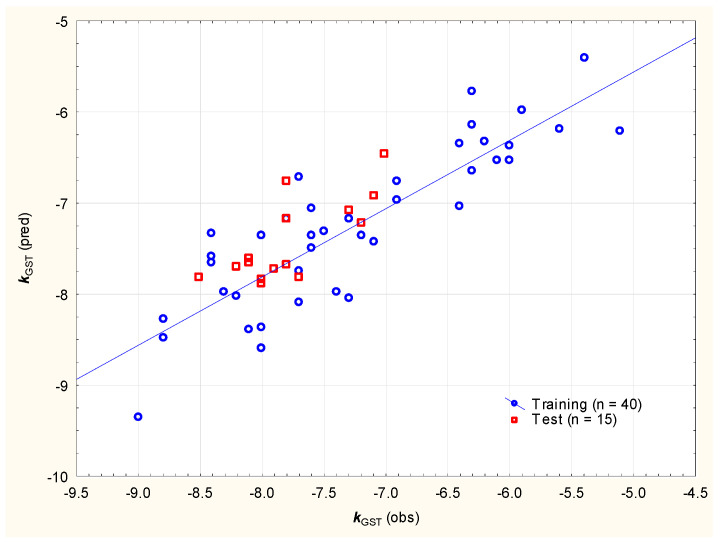
*k*_GST_ values calculated according to Equation (7) vs. expected.

The affinities of studied sunscreens and known/newly developed GST inhibitors for the placental GST enzyme can be described using Equation (7) (Figure 8):

*k*_GST_ = 3.755 (±2.655) + 0.111 (±0.028) ***nHA*** − 0.112 (±0.014) ***nRig*** + 2.081 (±0.576) ***MDCK*** (n = 40, R^2^ = 0.751, R^2^_adj._ = 0.730, F = 36.1, *p* < 0.01, RMSEP = 0.453, RMSECV = 0.629) (7)

According to Equation (7), the factors that facilitate strong ligand–GST enzyme binding are the high number of rigid (non-flexible) bonds (***nRig***), the lower number of H-bond acceptors (***nHA*** = sum of N and O atom counts) and the lower Madin–Darby canine kidney cells’ (***MDCKs***) logarithmic permeability (https://admetlab3.scbdd.com/, accessed on 8 December 2024). Apparent permeability values obtained on MDCK cell monolayers are used for screening of compounds in the context of membrane permeability at early stages of drug discovery [60]; it is often assumed that a high passive MDCK permeability ***P***_app_ is >20 × 10^−6^ cm/s, a medium permeability is 2 to 20 × 10^−6^ cm/s, and a low permeability is <2 × 10^−6^ cm/s. The independent variables in Equation (7) are introduced in the following order: ***nRig***, ***nHA*** and ***MDCK***; the ligands’ rigidity (***nRig***) accounts for ca. 58% of the total *k*_GST_ variability. ***MDCK*** is a complex variable, accounting for the molecules’ size (molecular weight, van der Waals volume) and polarity (topological polar surface area), and it appears that the same properties are, to some degree, responsible for ligand–enzyme binding.

**Figure 9:**
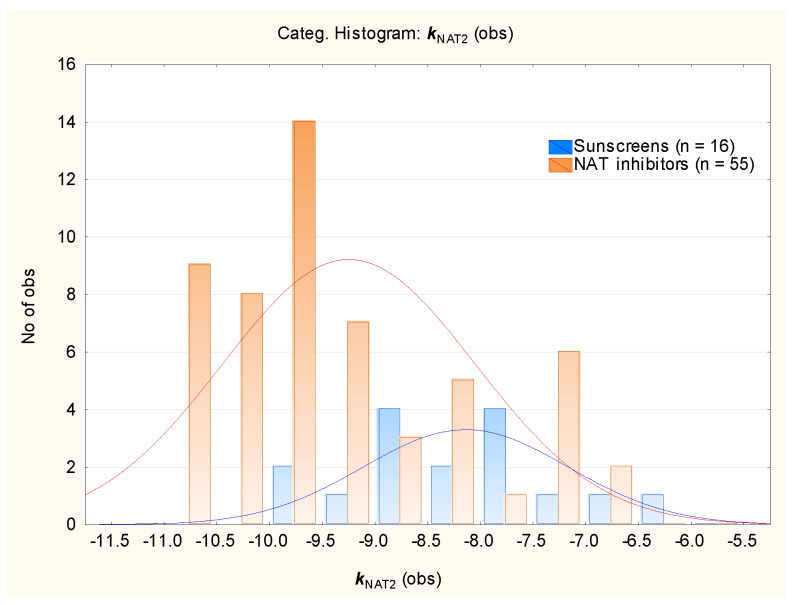
Histogram of *k*_NAT2_ values for the NAT inhibitors and studied sunscreens.

Similarly, the affinities of sunscreens for the NAT2 enzyme were compared to those obtained for several known or expected N-acetyltransferase inhibitors [61,62,63,64,65] (Figure 9); we determined that the mean *k*_NAT2_ for the sunscreens and inhibitors differed significantly (−8.2 and −9.3 for sunscreens and NAT inhibitors, respectively), but the *k*_NAT2_ values (provided in Supplementary Materials) for both groups of compounds are within similar ranges (Figure 9).

**Figure 10:**
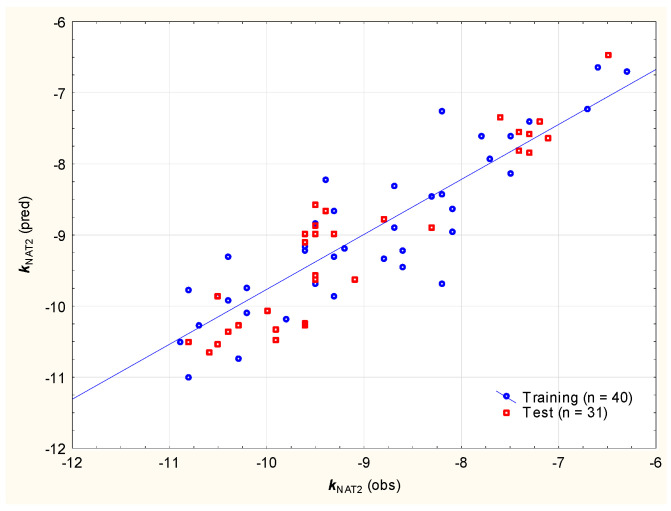
***k***_NAT2_ values calculated according to Equation (8) vs. expected.

The affinities of studied sunscreens and known/newly developed NAT inhibitors for the placental NAT2 enzyme can be described using Equation (8) (Figure 10), involving rigidity (***nRig***, accounting for almost 38% of total variability) and the lipophilicity descriptors log ***D*** and log ***P*** (https://admetlab3.scbdd.com/, accessed on 8 December 2024).

*k*_NAT2_ = −5.118 (±0.419) − 0.149 (±0.018) ***nRig*** − 1.148 (±0.176) log D + 0.591 (±0.076) log ***P*** (n = 40, R^2^ = 0.773, R^2^_adj._ = 0.754, F = 40.8, *p* < 0.01, RMSEP = 0.450, RMSECV = 0.804) (8)

Finally, the similarities and differences between the electrotopological state indices for the studied sunscreens and GST/NAT inhibitors were analyzed (Supplementary Materials). It was established that for the majority of the electrotopological state indices, there are no statistically significant differences between the sunscreens and the inhibitors. The basic molecular properties of sunscreens and enzyme inhibitors (https://admetlab3.scbdd.com/, accessed on 8 December 2024) were also compared (Supplementary Materials). It was found that for the GST inhibitors and 16 sunscreens, molecular weight (***MW***), volume (***Vol***), H-bond donor count (***nHD***), H-bond acceptor count (***nHA***), formal charge (***fChar***), rigid bond count (***nRig***), rotatable bond count (***nRot***), flexibility = ***nRot***/***nRig*** (***Flex***), stereocenter count (***nStereo***), fraction of sp^3^ carbons (***F***_Csp3_) and topological surface area (***TPSA***) are similar, with no statistically significant differences. For the NAT inhibitors and 16 sunscreens, the differences are more pronounced, with only ***MW***, ***Vol***, ***nHA***, ***fChar*** and ***nStereo*** showing a high degree of similarity. This observation is in line with the results reported above (Table 2)—only 4 out of 16 sunscreens (vs. 13 out of 16 for the GST enzyme) exhibit very strong affinity for the NAT2 enzyme.

## 3. Materials and Methods

### 3.1. Compounds

Compounds ***1*** to ***54***, whose experimental placenta permeability data are available and were used to generate qualitative models described in Section 2, were obtained from [66] and are listed in Supplementary Materials. These compounds were divided into two sets: a training set (compounds ***1*** to ***40***) and a test set (compounds ***41*** to ***54***). Binary High/Low placenta permeability indices (PL1/PL0, respectively) for compounds ***1*** to ***54*** were obtained from [67] (Table S1, Supplementary Materials). Compounds ***55*** to ***93*** (GST enzyme inhibitors) and ***94*** to ***148*** (NAT enzyme inhibitors) were obtained from sources in the literature, as stated in Section 2.3.

### 3.2. Calculated Descriptors

Molecular weight (***M****_w_*), heavy atom count (*#**HvAt***), aromatic heavy atom count (*#**ArHvAt***), fraction of sp^3^ carbons (***F***_Csp3_), rotatable bond count (#***FRB***), hydrogen bond donor count (#***HD***), hydrogen bond acceptor count (#***HA***), molar refractivity (***MR***), octanol–water partition coefficient (***XLOGP3***) and topological polar surface area (***TPSA***) were calculated using SwissADME software (http://www.swissadme.ch/, accessed on 15 October 2024) [68]. Mordred 2D and 3D descriptors [69] for 16 sunscreens and compounds ***1*** to ***54*** were calculated using the OCHEM platform (https://ochem.eu, accessed on 15 October 2024) with Baloon optimization (3D) [70]. OEState descriptors [51,52] for 16 sunscreens and compounds ***55*** to ***148*** were generated using OCHEM platform, and the basic physicochemical descriptors for these compounds were calculated using ADMETlab3.0 software (https://admetlab3.scbdd.com/, accessed on 5 December 2024). All the relevant molecular descriptors of compounds ***1*** to ***148*** and 16 sunscreens are provided in Supplementary Materials (the descriptors actually used in classification models are highlighted in Table S1).

### 3.3. Placenta Permeability Classification Models

A pool of 1836 Mordred and SwissADME descriptors was reduced using partial least squares (PLS) (Statistica v. 13.3, Statsoft, Kraków, Poland) regression (the NIPALS algorithm with auto-scaling). Variables with zero variation or those that are very strongly co-linear were removed automatically. The remaining variables were evaluated using their variable importance in projection (VIP) values (those with VIP < 1 were skipped, and the PLS procedure was repeated) [71].

General discriminant analysis (GDA) models were generated using Statistica v. 13.3 by StatSoft Polska, Kraków, Poland, in forward stepwise regression mode. Multilayer perceptron (MLP) artificial neural networks (ANNs) were generated using Statistica v. 13.3 (classification mode, Automated Network Search—ANS module, 1000 networks to train, 50 networks to retain). The possible neuron activation functions were as follows: identity, logistic, hyperbolic tangent and exponential. The BFGS (Broyden–Fletcher–Goldfarb–Shanno) algorithm and the sum of squares (SOS) error function were used to train the network. The results of classification (class memberships of compounds ***1*** to ***54*** and 16 studied sunscreens) are in in Table S1 (Supplementary Materials).

### 3.4. Molecular Docking

The structures and data files (sdf) of studied sunscreens were downloaded from the PubChem database (https://pubchem.ncbi.nlm.nih.gov/, accessed on 15 October 2024). Their energies were minimized in PyRx v. 0.8 software (https://pyrx.sourceforge.io/, accessed on 15 October 2024) [72] using universal force field at 400 steps; then, the compounds were converted to AutoDock ligands (pdbqt) and used for the molecular docking analysis.

The 3D X-ray crystallographic structures of two enzymes active in the human placenta—glutathione s-transferase (GST (ID: 1LJR)) and human N-acetyltransferase 2 (NAT2 (ID: 2PFR))—were obtained as pdb files from the protein data bank (https://www.rcsb.org, accessed on 10 October 2024). The removal of the interfering crystallographic water molecules and previously studied ligands was performed with Discovery Studio Visualizer (Biova, Dassault Systèmes, Paris, France). The possible active sites of the enzymes were identified based on earlier data regarding other chemicals’ binding to GST and NAT2 enzymes [42]. The docking of studied ligands on the enzymes’ active sites was performed in PyRx v. 0.8 [72] using the grid box with the following dimensions: for the GST enzyme, center x: 12.389, center y: 67.111, center z: 2.306, size x: 22.139, size y: 29.221, size z: 33.415; for the NAT2 enzyme, center x: 8.326, center y: 38.309, center z: 65.486, size x: 24.365, size y: 32.644, size z: 24.861. The binding poses of the protein–ligand complexes were generated, and their binding affinity results ***k*** (kcal/mol) were obtained (Table 2). Molecular enzyme–ligand interactions were visualized in 2D using Discovery Studio Visualizer (Figure S1, Supplementary Materials).

### 3.5. Multiple Linear Regression Models (MLRs) of Ligands’ Affinity for GST and NAT2 Enzymes

MLR models were generated using Statsoft v. 13.3; OEstate descriptors with zero variability (***HALOG***, ***P***) were identified and removed manually based on their standard deviation values (SD = 0) for studied sunscreens. The suitable descriptors for MLR models were selected using stepwise regression in forward mode. Equations (4) and (5) were subjected to 4-fold cross-validation (RMSECV) and Equations (6) and (7) were validated using 4-fold cross-validation (RMSECV) and external test sets of compounds (RMSEPs) [73].

## 4. Conclusions

Sunscreens used to protect skin, hair and materials are usually able to cross the placenta very easily, mostly by passive diffusion—with the exception of bulky and highly lipophilic compounds such as DHHB, OCR, ET and DOBT, whose passage across the placenta appears to be difficult.

Some organic sunscreens can be suspected of interfering with enzymes present in the placenta which are responsible for deactivation of undesired xenobiotics. Such enzymes include, for example, glutathione *S*-transferases (GSTs). Enzymes from this family can facilitate detoxifcations of many endogenous compounds and xenobiotics by conjugation with reduced glutathione. Another enzymatic tool capable of protecting the fetus against toxines is human N-acetyltransferase 2 (NAT2), reponsible for deactivation of arylamine and hydrazine compounds. It was established that sunscreens (including those that are not able to easily cross the placenta, such as DOBT or ET) have a potential to bind to GST and NAT2 enzymes—mainly by hydrogen bonds, van der Waals, π-π, alkyl, π-alkyl and π-sulfur interactions that all contribute to the stability of enzyme–ligand complexes. Theoretical predictions of sunscreens’ affinity for GST and NAT2 enzyme targets could, potentially, be confirmed by in vitro experiments, e.g., using biomimetic chromatography on immobilized protein stationary phases capable of mimicking protein–ligand interactions encountered in the living organism. Investigations involving the use of commercially available protein stationary phases *in lieu* of sorbents containing immobilized actual GST or NAT2 proteins are underway.

At present, there is a lot of interest among consumers in sunscreens’ safety during pregnancy and nursing [74], and there are no strict recommendations for females at these stages in life to use specific UV filters [75]. There is, however, some evidence (based mainly on in vitro studies or animal data) that certain sunscreens (including BP-3, OCR, BMDM, EHMC, MBC, OS and HMS) should be avoided by pregnant and breast-feeding women [76,77,78], and new facts contraindicating the use of these substances during pregnancy are being discovered—for example, there are some early reports that BP-3 may be associated with Hirschsprung’s disease [76,79,80]. It seems that there are several suscreens in the studied group that are, potentially, very good placenta penetrators and, at the same time, likely ligands for GST and/or NAT2 enzymes, which confirms the notion that, during pregnancy, organic sunscreens should be used with caution, and new evidence against their excessive use is likely to appear.

## Figures and Tables

**Table 1:** Mean values of physicochemical parameters and filters for PL0 and PL1 compounds (parameters significantly different between the classes are highlighted).

Variable	PL0 (n = 16)	PL1 (n = 54)
***M***_w_	**493.72**	**292.63**
#***HvAt***	**32.75**	**20.28**
#***ArHvAt***	**11.06**	**7.35**
***F***_Csp3_	0.47	0.43
#***HRB***	**9.94**	**4.96**
#***HA***	3.69	3.83
#***HD***	1.69	1.44
***MR***	**134.97**	**80.67**
***TPSA***	70.41	70.65
***XLOGP3***	**6.42**	**2.40**
***nAcid***	0.00	0.20
***nBase***	0.25	0.43
***nSpiro***	0.00	0.09
***nBridgehead***	0.38	0.26
***nHetero***	**7.31**	**5.52**
***Lipinski***	**0.06**	**0.94**
***GhoseFilter***	**0.25**	**0.80**

**Table 2:** Binding affinities of studied sunscreens on GST and NAT2 proteins (binding affinities stronger than those for the reference ligands are highlighted).

	*k*_GST_ (kcal/mol)	*k*_NAT2_ (kcal/mol)
BMDM	**−7.50**	**−9.60**
BP-3	**−7.30**	−8.70
DHHB	**−6.90**	−8.20
PABA	−5.10	−6.30
EHDP	−6.00	−7.50
Et-PABA	−5.60	−6.70
PBSA	**−7.70**	**−9.50**
MBC	**−7.60**	**−8.80**
EHMC	**−6.30**	−7.80
IMC	**−6.30**	−7.70
OCR	**−7.80**	−8.70
ET	**−7.40**	−7.30
OS	**−6.20**	−8.20
HMS	**−7.60**	−8.70
DOBT	**−8.20**	−7.50
BP-4	**−8.00**	**−9.30**
Reference	−6.00	−8.70

**Table 3:** Main binding aminoacids of GST protein and types of interactions (values represent no. of cases out of 16 ligands).

GST	Leu A114	Leu A119	Val A36	Leu A35	Val A10	Gln A12	Met A236	Trp A115	Total
van der Waals	13	7	9	1	8	7	6	2	53
H-bond				1	1	2		6	10
π-σ		4		7					11
alkyl	2	5	5	7	5		3	3	30
π-alkyl									0
unfavorable donor–donor						1			1
π-π								3	3

**Table 4:** Main binding aminoacids of NAT2 protein and types of interactions (values represent no. of cases out of 16 ligands).

NAT2	Phe A217	Phe A93	Phe A37	Gly A126	Ser A125	Leu A288	Ser A129	Cys A68	Total
van der Waals	4	2	10	10	13	9	10		58
H-bond				1	2				3
π-sulfur								6	6
π-π	9	11	1						21
alkyl	2	2	4	1		6			15
π-σ		1				1			2

**Table 5:** Correlations between ***k***_NAT2_, ***k***_GST_ and physicochemical properties of 16 sunscreens (marked correlations are significant at *p* < 0.5000).

	*k*_GST_	*k*_NAT2_	*M*_w_	#*HvAt*	#*ArHvAt*	*F*_Csp3_	#*FRB*	#*HA*	*MR*	#*HD*	*TPSA*	*XLOGP3*
***k***_GST_	1.00	**0.72**	**−0.51**	**−0.50**	**−0.62**	−0.02	−0.27	**−0.51**	−0.28	−0.48	−0.43	−0.40
***k***_NAT2_	**0.72**	1.00	0.10	0.11	−0.08	0.13	0.29	0.03	0.24	0.13	0.12	0.15
***M***_w_	**−0.51**	0.10	1.00	**1.00**	**0.90**	0.35	**0.95**	**0.90**	**0.65**	**1.00**	**0.82**	**0.94**
#***HvAt***	**−0.50**	0.11	**1.00**	1.00	**0.89**	0.36	**0.95**	**0.88**	**0.64**	**1.00**	**0.80**	**0.94**
#***ArHvAt***	**−0.62**	−0.08	**0.90**	**0.89**	1.00	−0.07	**0.78**	**0.90**	**0.76**	**0.87**	**0.89**	**0.73**
***F***_Csp3_	−0.02	0.13	0.35	0.36	−0.07	1.00	0.45	0.05	−0.24	0.40	−0.15	**0.58**
#***FRB***	−0.27	0.29	**0.95**	**0.95**	**0.78**	0.45	1.00	**0.83**	**0.55**	**0.96**	**0.72**	**0.95**
#***HA***	**−0.51**	0.03	**0.90**	**0.88**	**0.90**	0.05	**0.83**	1.00	**0.80**	**0.86**	**0.95**	**0.74**
***MR***	−0.28	0.24	**0.65**	**0.64**	**0.76**	−0.24	**0.55**	**0.80**	1.00	**0.61**	**0.93**	0.43
#***HD***	−0.48	0.13	**1.00**	**1.00**	**0.87**	0.40	**0.96**	**0.86**	**0.61**	1.00	**0.77**	**0.96**
***TPSA***	−0.43	0.12	**0.82**	**0.80**	**0.89**	−0.15	**0.72**	**0.95**	**0.93**	**0.77**	1.00	**0.60**
***XLOGP3***	−0.40	0.15	**0.94**	**0.94**	**0.73**	**0.58**	**0.95**	**0.74**	0.43	**0.96**	**0.60**	1.00

## Data Availability

Data generated in this study can be found in this manuscript.

## Supplementary Materials

The following supporting information can be downloaded at: https://www.mdpi.com/article/10.3390/molecules29245836/s1: Table S1: Key molecular descriptors, observed and predicted PL0/PL1 classification of studied compounds; Figure S1: 2D plots of protein-ligand interactions; Supplementary Materials molecular descriptors 08.12.2024.xlsx.
