# Supplementary material for: Organic Sunscreens—Is Their Placenta Permeability the Only Issue Associated with Exposure During Pregnancy? In Silico Studies of Sunscreens’ Placenta Permeability and Interactions with Selected Placental Enzymes
Source: Molecules. 2024 Dec 11;29(24):5836. doi: 10.3390/molecules29245836 (PMC11728689; doi:10.3390/molecules29245836)

Figure S1. 2D plots of protein-ligand interactions

**BP3-GST**

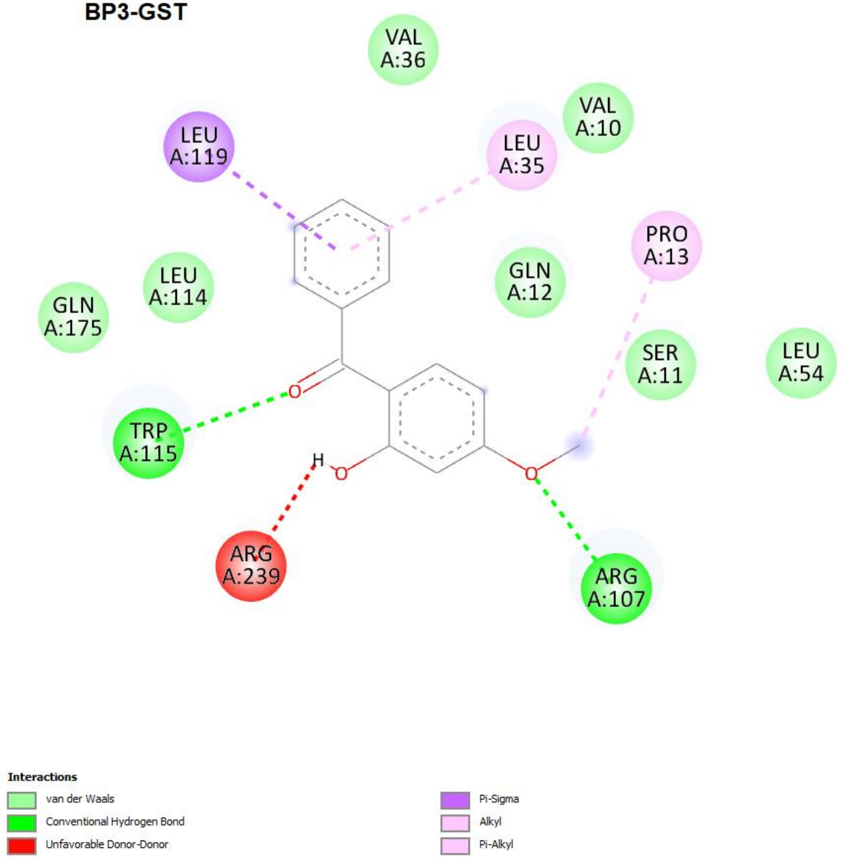


**BP3-NAT2**

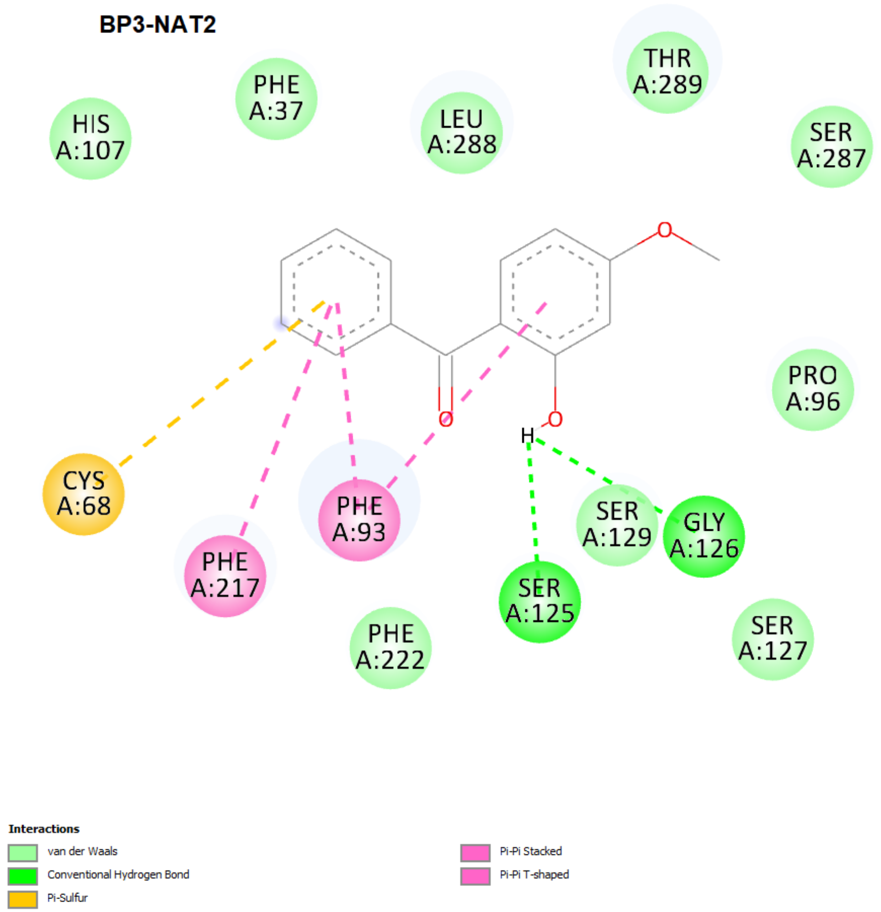

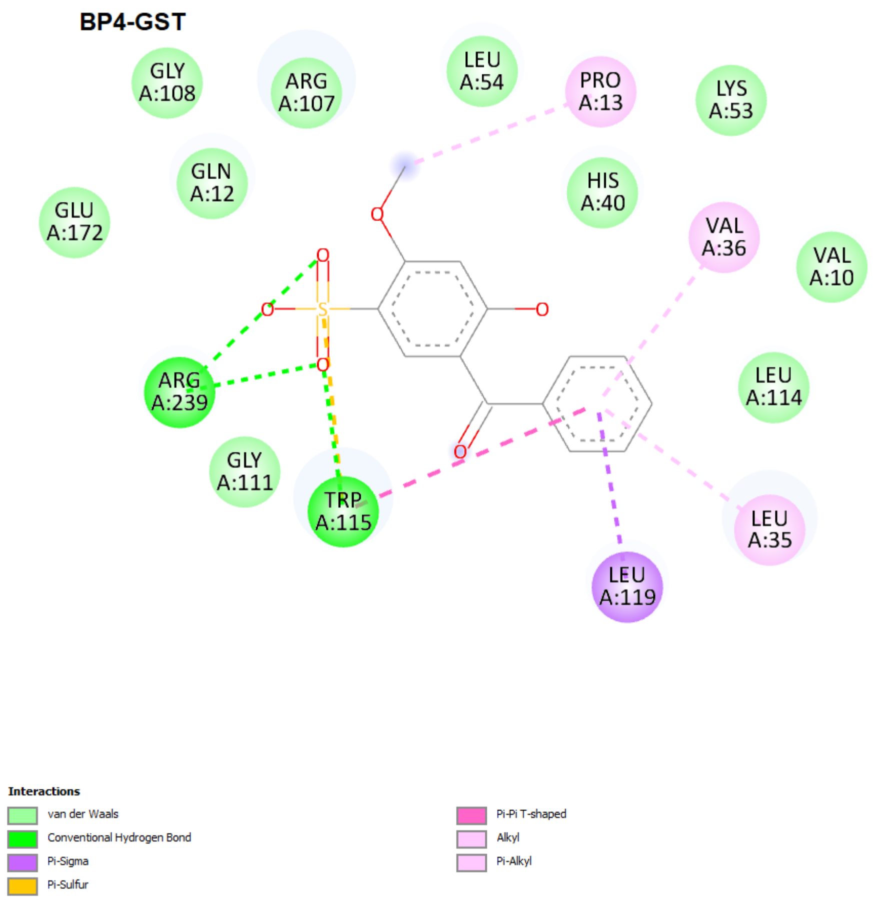
BP4-GST
GLY A:108
ARG A:107
LEU A:54
PRO A:13
LYS A:53
GLN A:12
GLU A:172
HIS A:40
VAL A:36
VAL A:10
ARG A:239
LEU A:114
GLY A:111
TRP A:115
LEU A:35
LEU A:119
Interactions
van der Waals
Conventional Hydrogen Bond
Pi-Sigma
Pi-Sulfur
Pi-Pi T-shaped
Alkyl
Pi-Alkyl

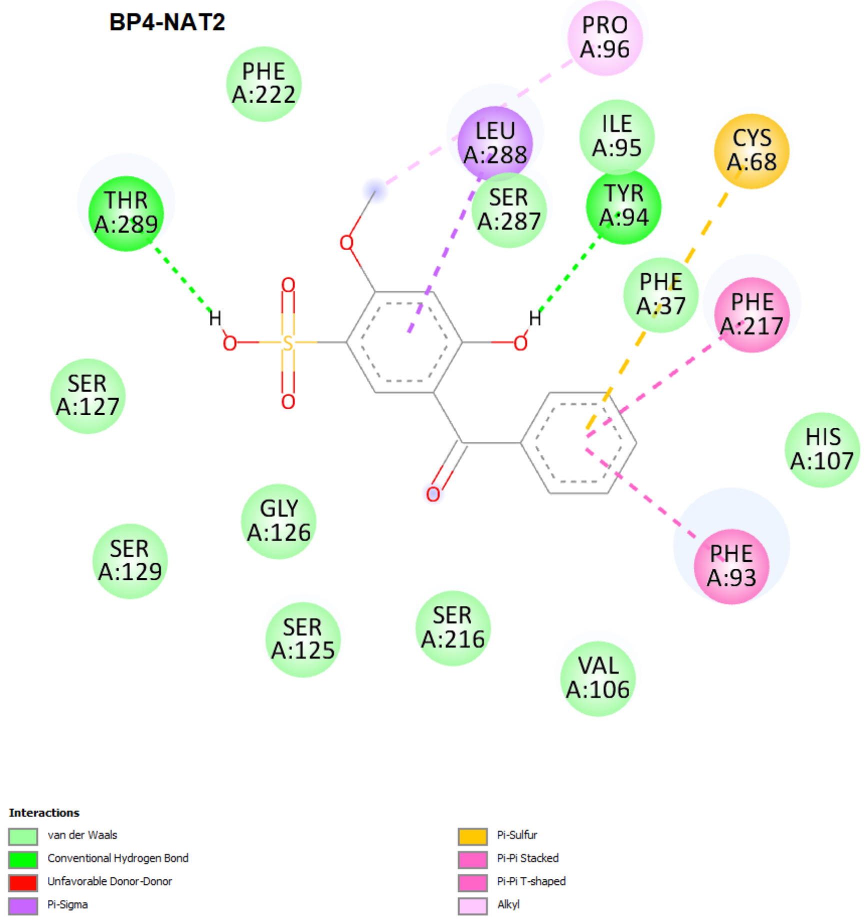
BP4-NAT2
PRO A:96
PHE A:222
LEU A:288
ILE A:95
CYS A:68
THR A:289
SER A:287
TYR A:94
PHE A:37
PHE A:217
SER A:127
HIS A:107
GLY A:126
SER A:129
PHE A:93
SER A:125
SER A:216
VAL A:106
Interactions
van der Waals
Conventional Hydrogen Bond
Unfavorable Donor-Donor
Pi-Sigma
Pi-Sulfur
Pi-Pi Stacked
Pi-Pi T-shaped
Alkyl

DHHB-GST

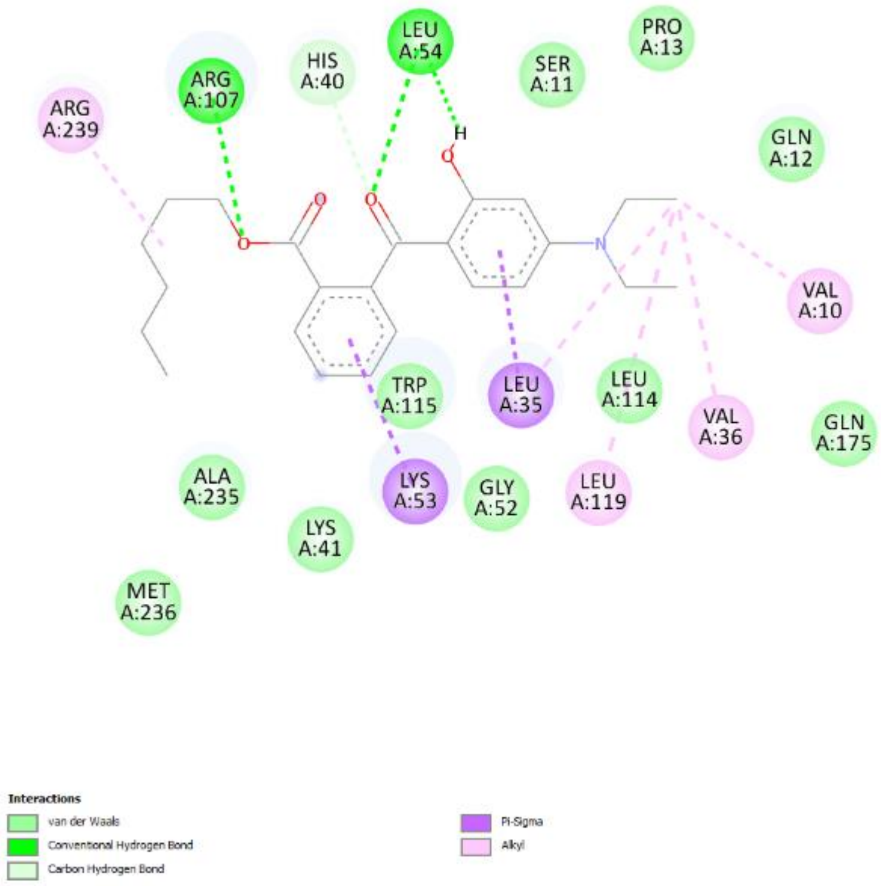


DHHB-NAT2

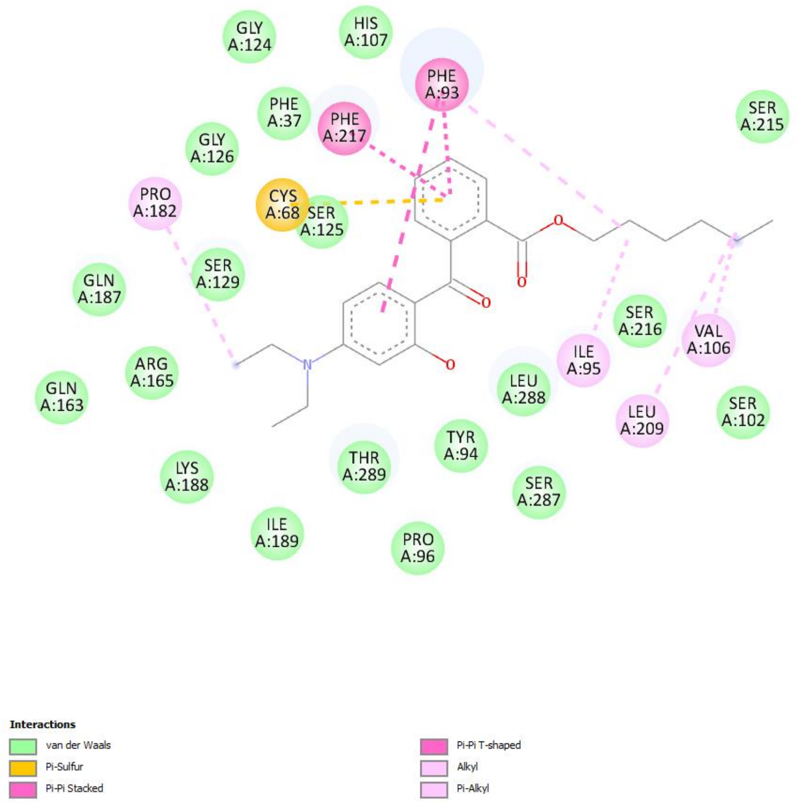

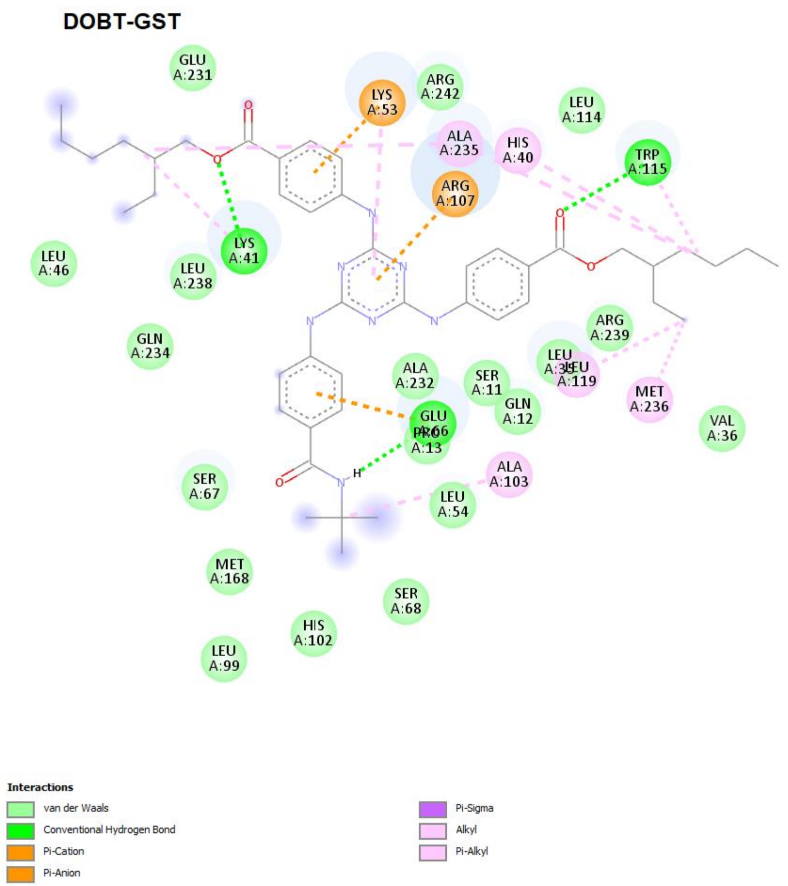
DOBT-GST
GLU A:231
LYS A:53
ARG A:242
LEU A:114
ALA A:235
HIS A:40
TRP A:115
ARG A:107
LEU A:46
LYS A:41
LEU A:238
GLN A:234
ARG A:239
ALA A:232
SER A:11
GLN A:12
LEU A:119
MET A:236
VAL A:36
GLU A:13
SER A:67
ALA A:103
LEU A:54
MET A:168
SER A:68
HIS A:102
LEU A:99
Interactions
van der Waals
Conventional Hydrogen Bond
Pi-Cation
Pi-Anion
Pi-Sigma
Alkyl
Pi-Alkyl

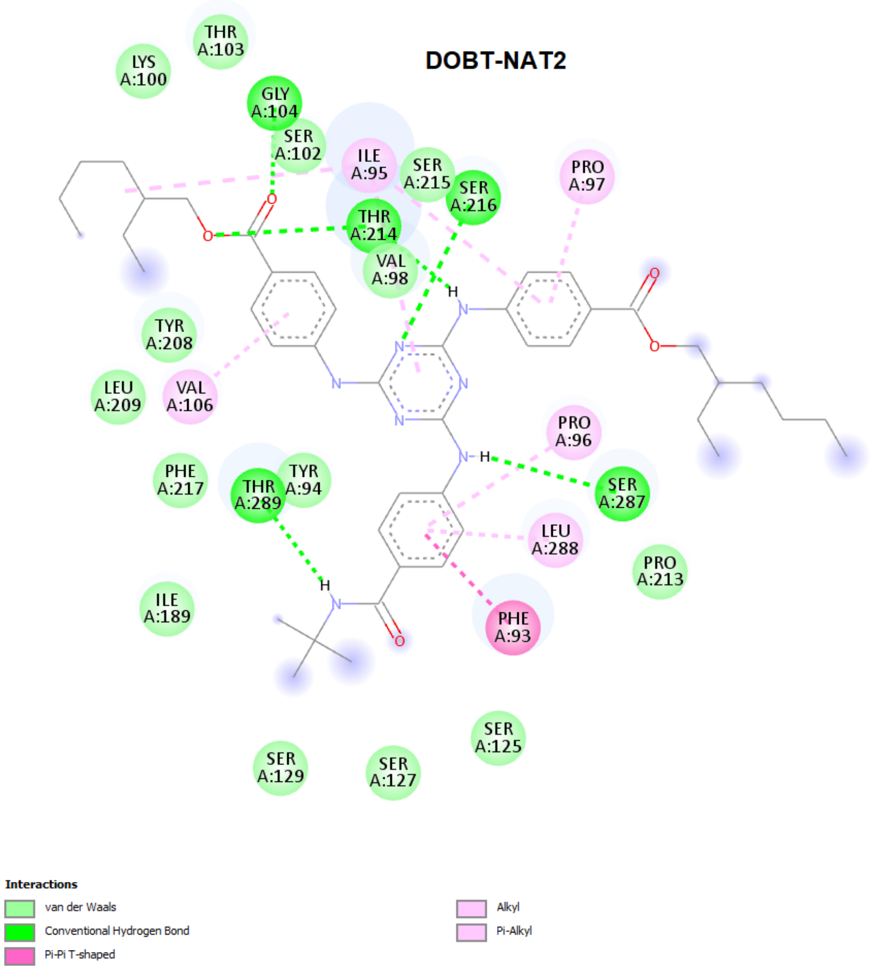
DOBT-NAT2
THR A:103
LYS A:100
GLY A:104
SER A:102
ILE A:95
SER A:215
SER A:216
PRO A:97
THR A:214
VAL A:98
TYR A:208
LEU A:209
VAL A:106
PRO A:96
PHE A:217
TYR A:94
THR A:289
SER A:287
LEU A:288
PRO A:213
ILE A:189
PHE A:93
SER A:125
SER A:129
SER A:127
Interactions
van der Waals
Conventional Hydrogen Bond
Pi-Pi T-shaped
Alkyl
Pi-Alkyl

**EHDP-GST**

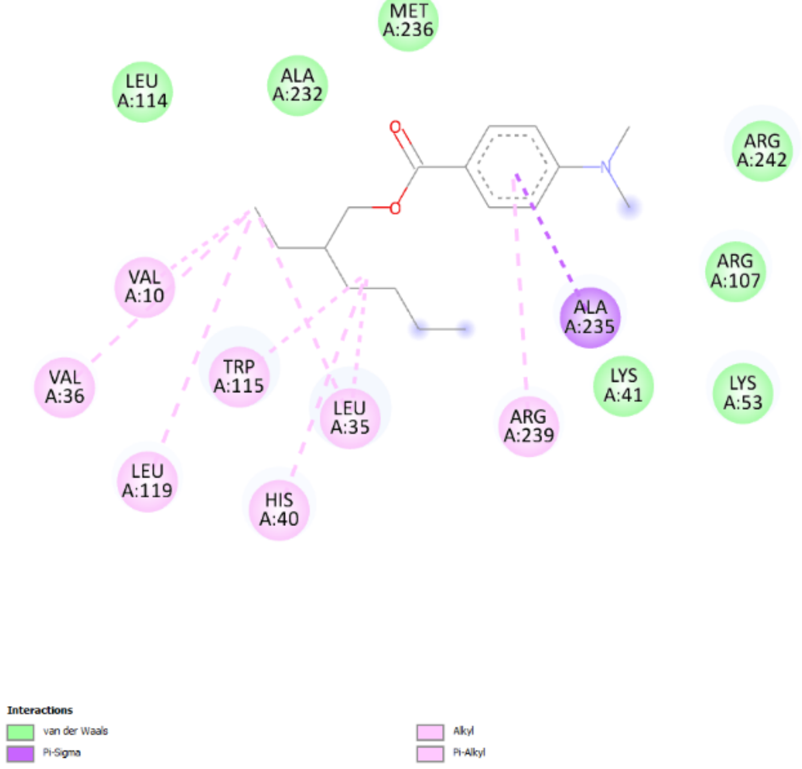


**EHDP-NAT2**

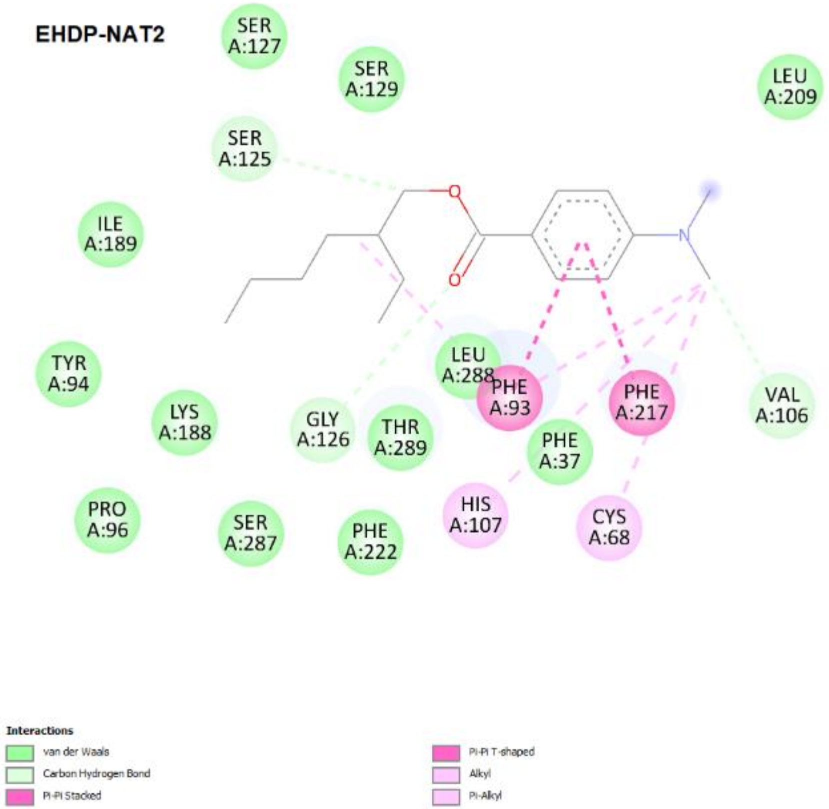

EHMC-GST

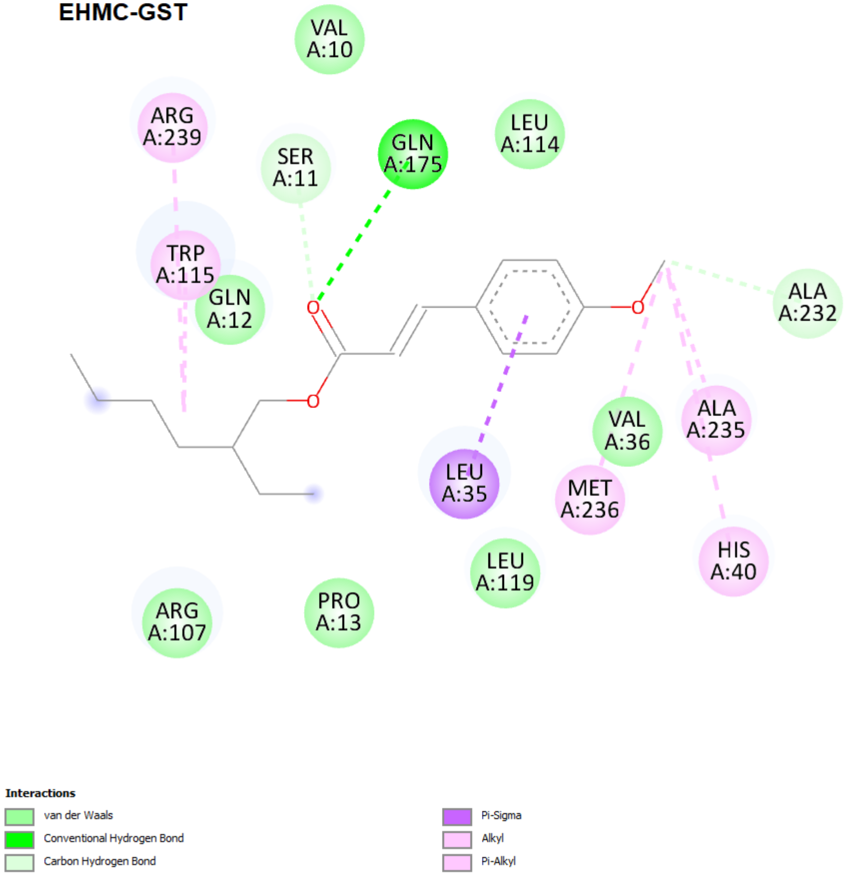


EHMC-NAT2

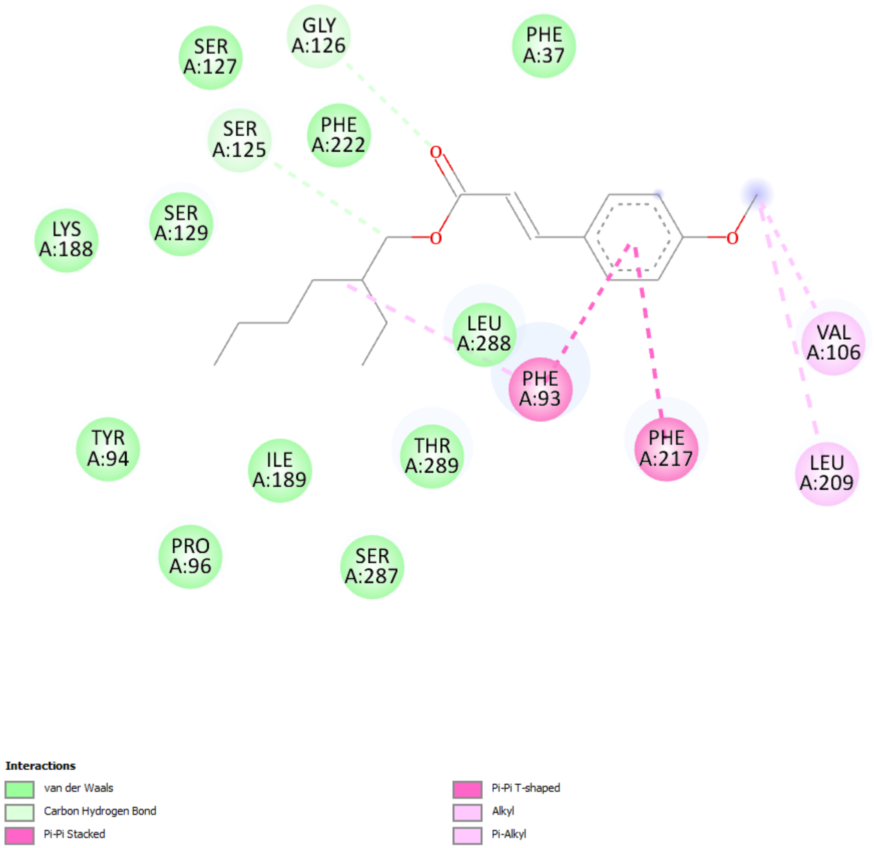

ET-GST

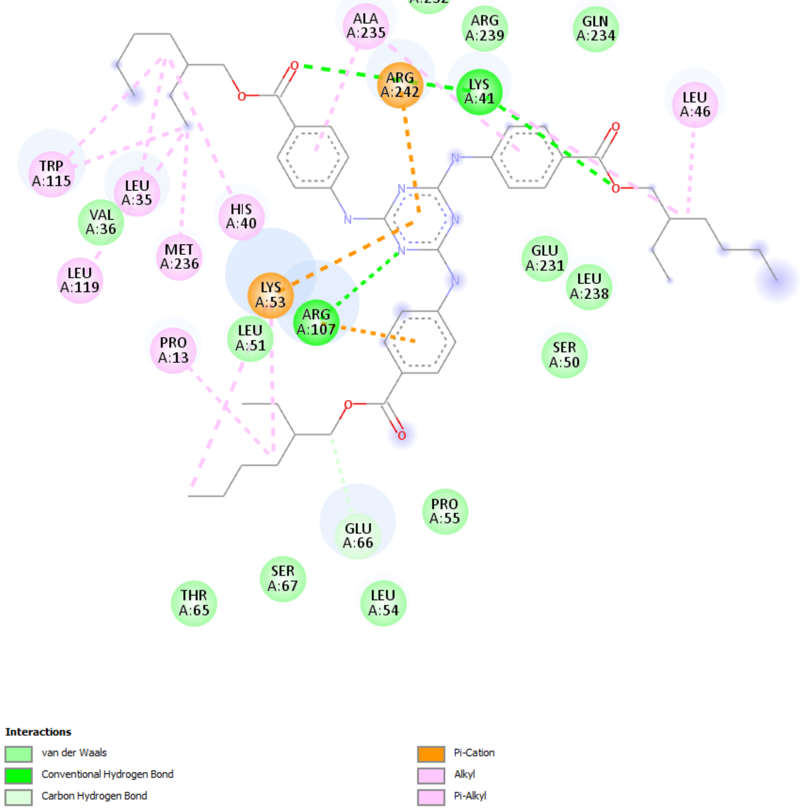


ET-NAT2

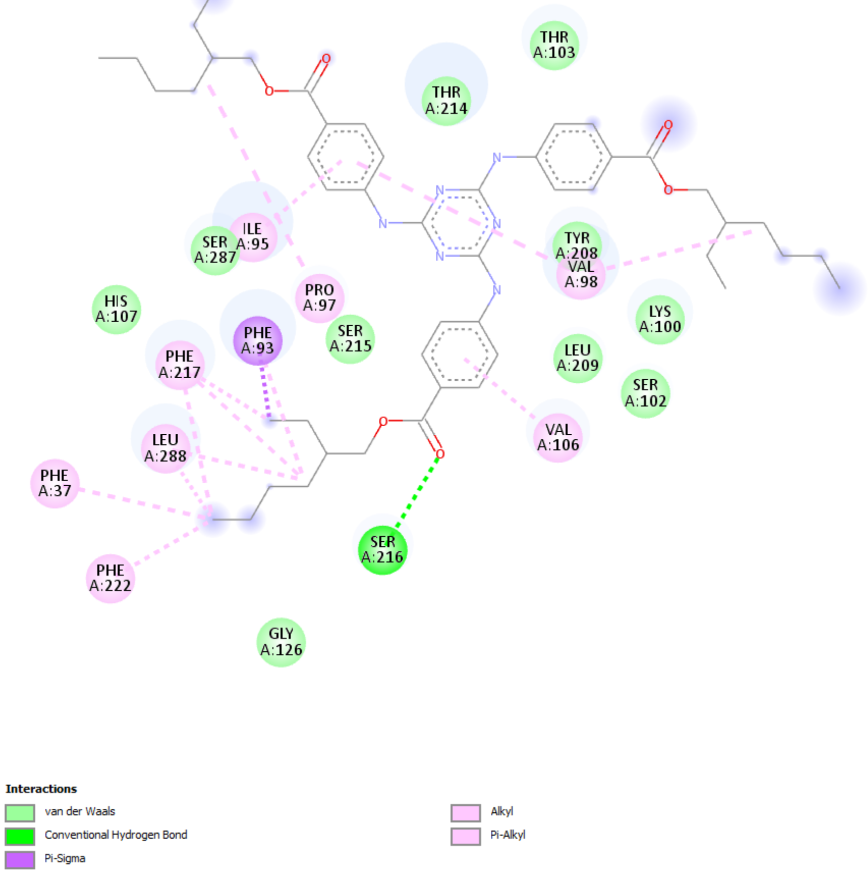

**Et-PABA-GST**

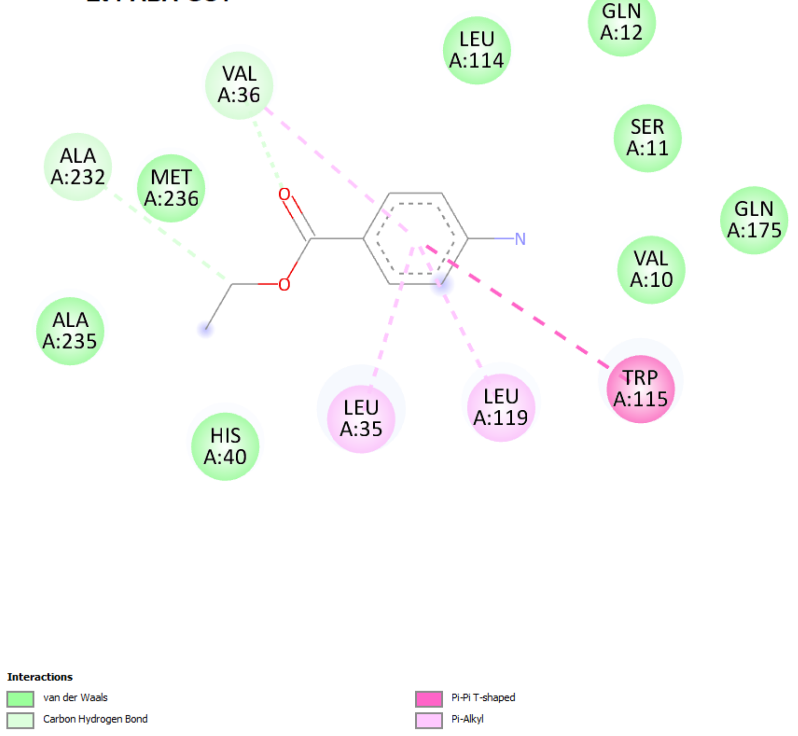


**EtPABA-NAT2**

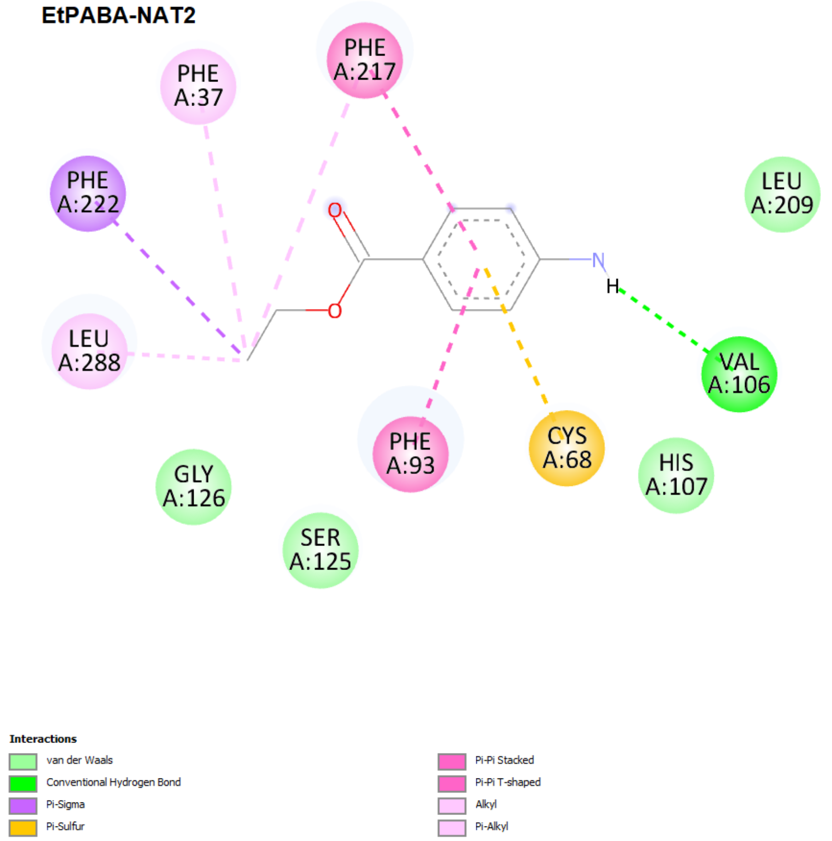

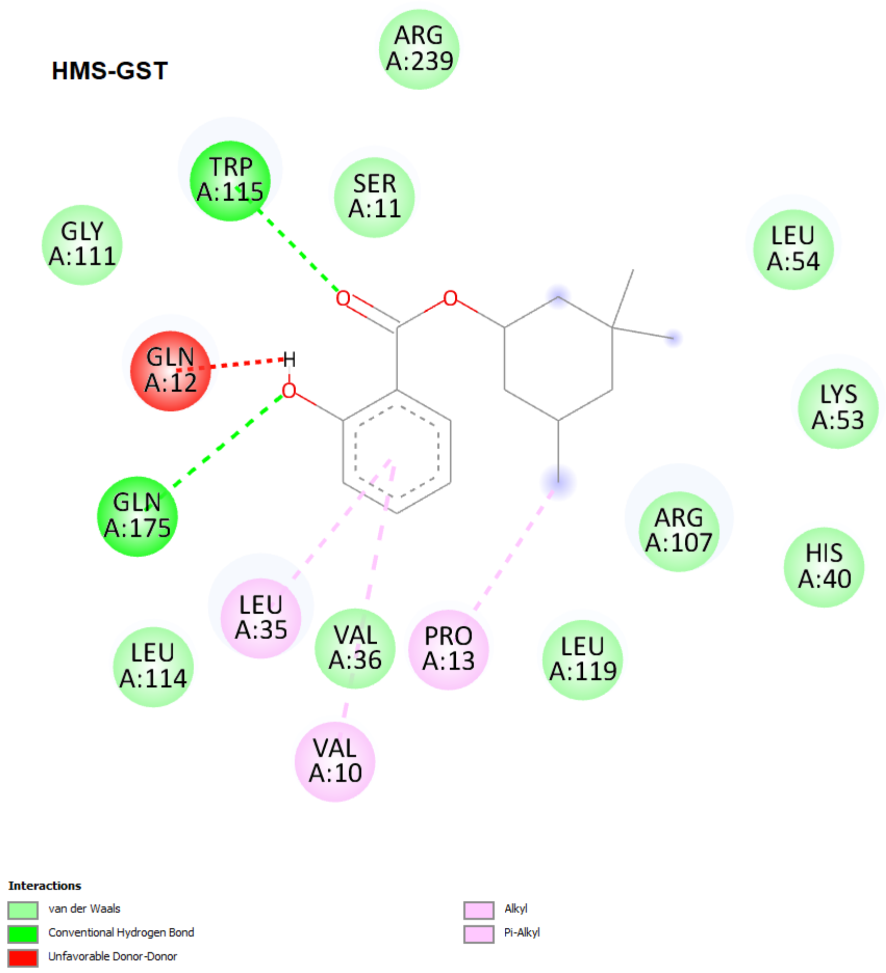
HMS-GST
ARG A:239
TRP A:115
SER A:11
GLY A:111
LEU A:54
GLN A:12
LYS A:53
GLN A:175
ARG A:107
HIS A:40
LEU A:35
VAL A:36
PRO A:13
LEU A:119
LEU A:114
VAL A:10
Interactions
van der Waals
Conventional Hydrogen Bond
Unfavorable Donor-Donor
Alkyl
Pi-Alkyl

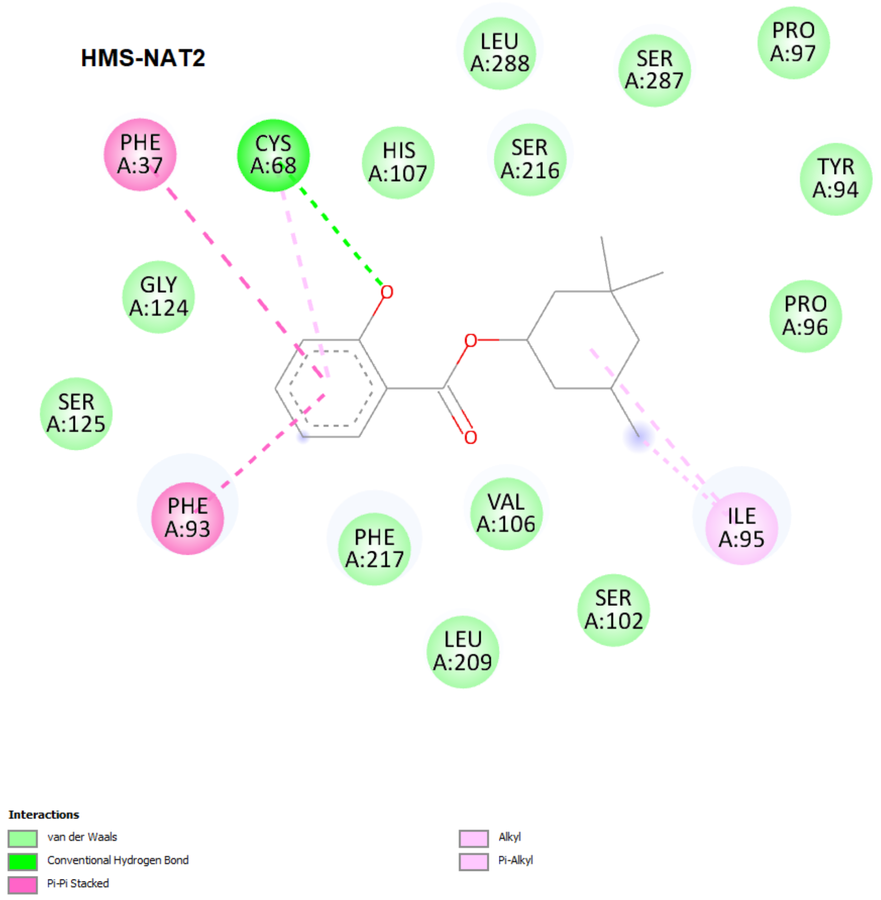
HMS-NAT2
LEU A:288
SER A:287
PRO A:97
PHE A:37
CYS A:68
HIS A:107
SER A:216
TYR A:94
GLY A:124
PRO A:96
SER A:125
PHE A:93
PHE A:217
VAL A:106
ILE A:95
SER A:102
LEU A:209
Interactions
van der Waals
Conventional Hydrogen Bond
Pi-Pi Stacked
Alkyl
Pi-Alkyl

IMC-GST

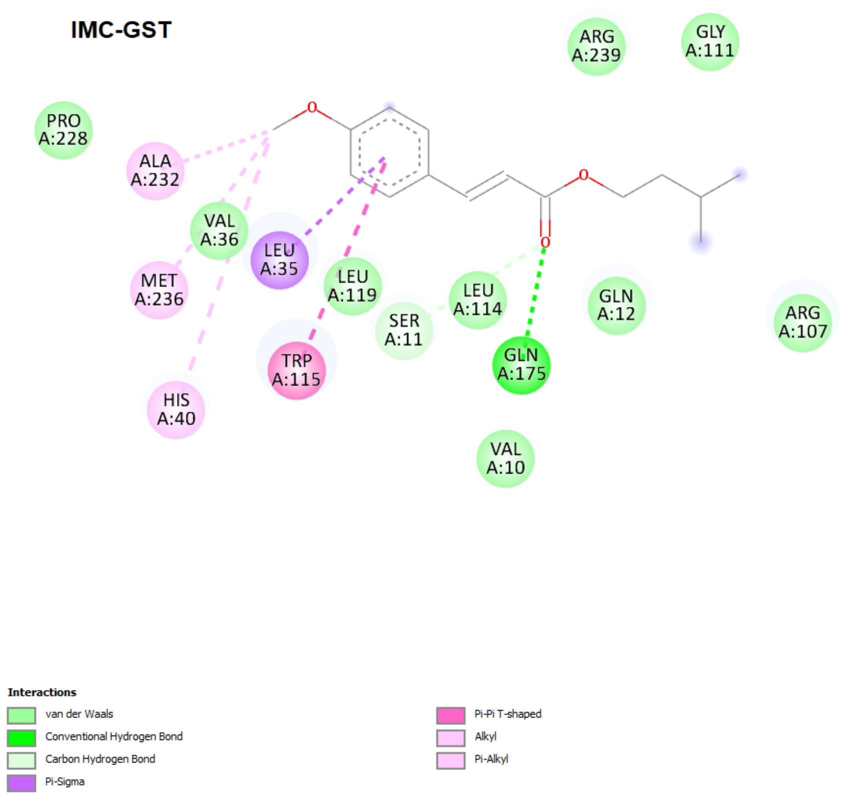


IMC-NAT2

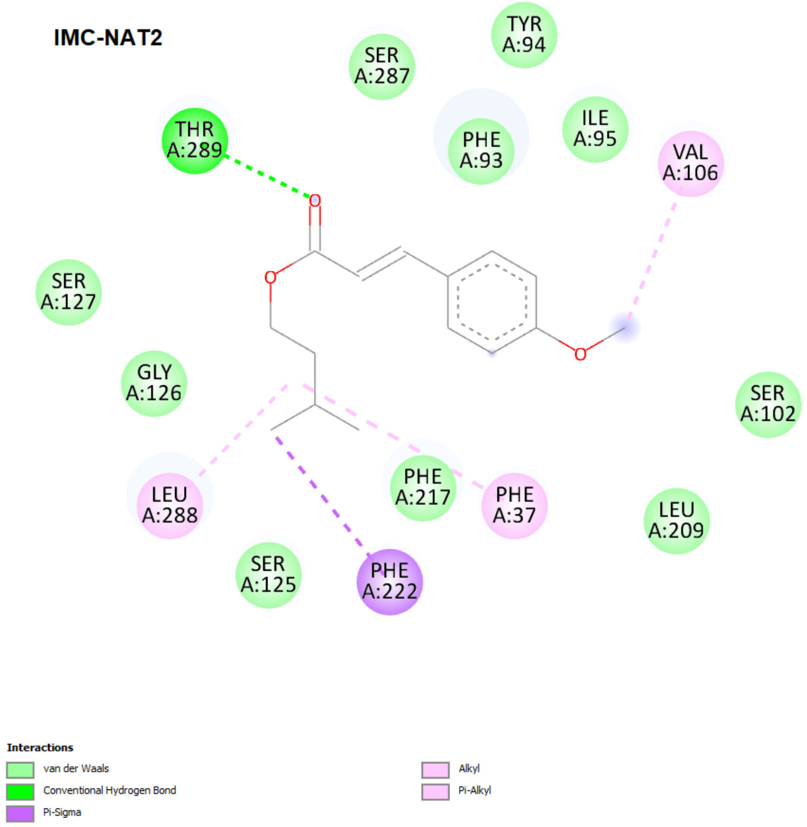

**MBC-GST**

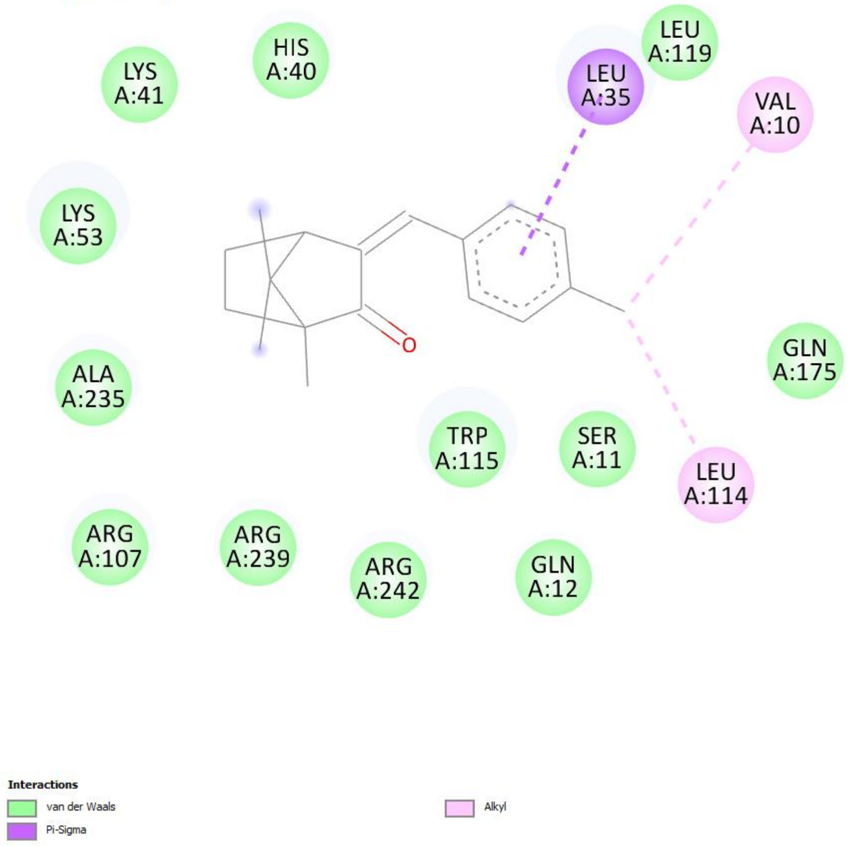


**MBC-NAT2**

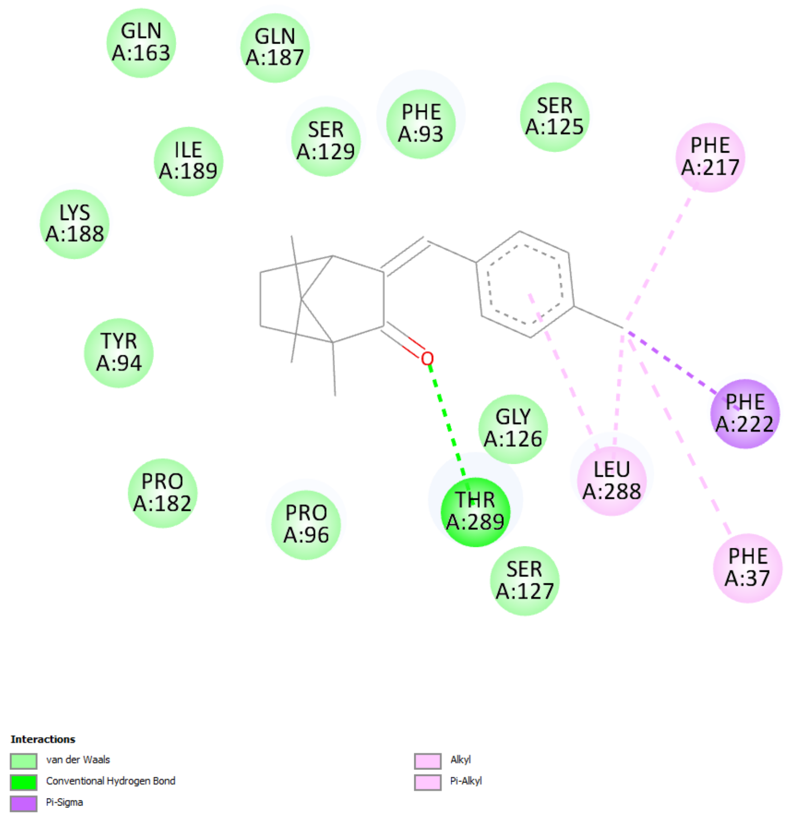

OCR-GST

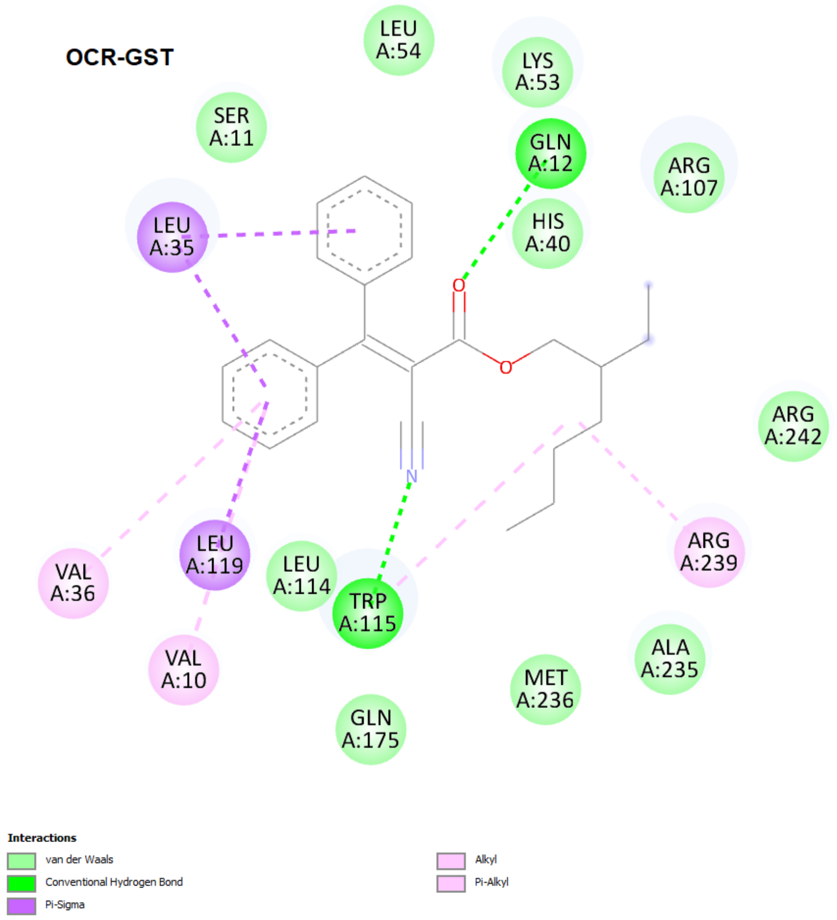


OCR-NAT2

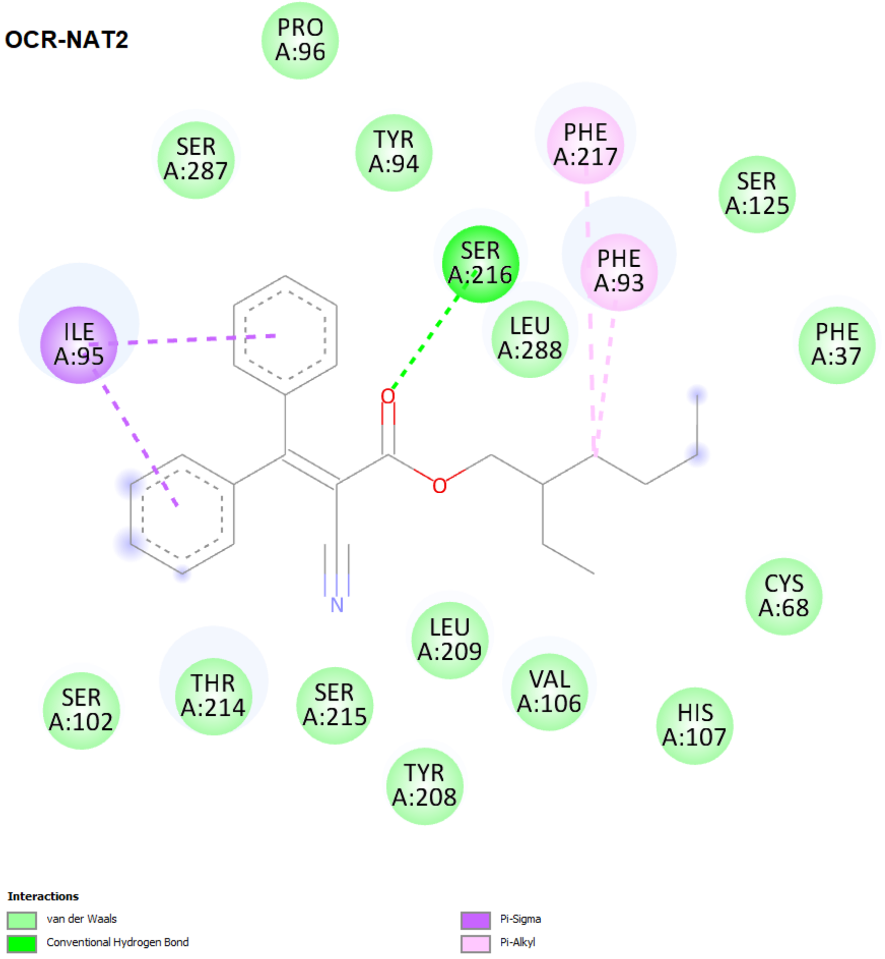

**OS-GST**

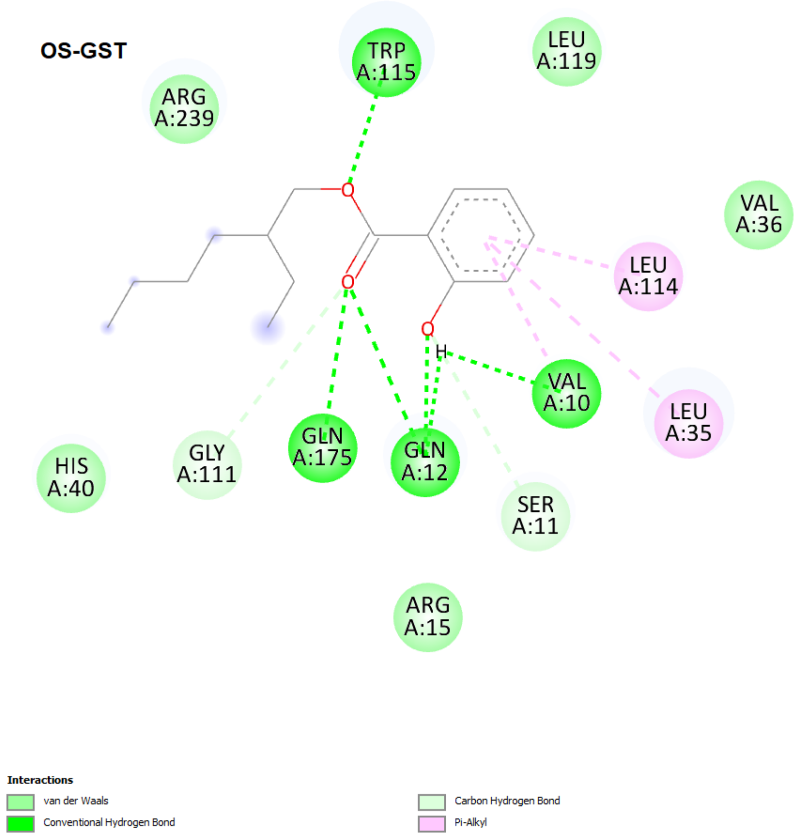


**OS-NAT2**

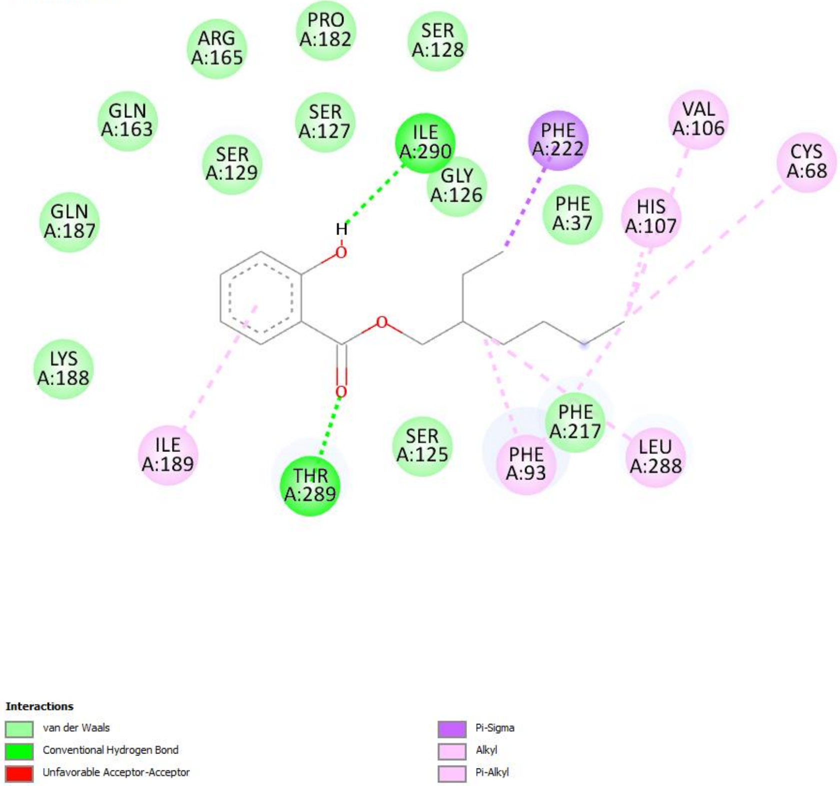

**PABA-GST**

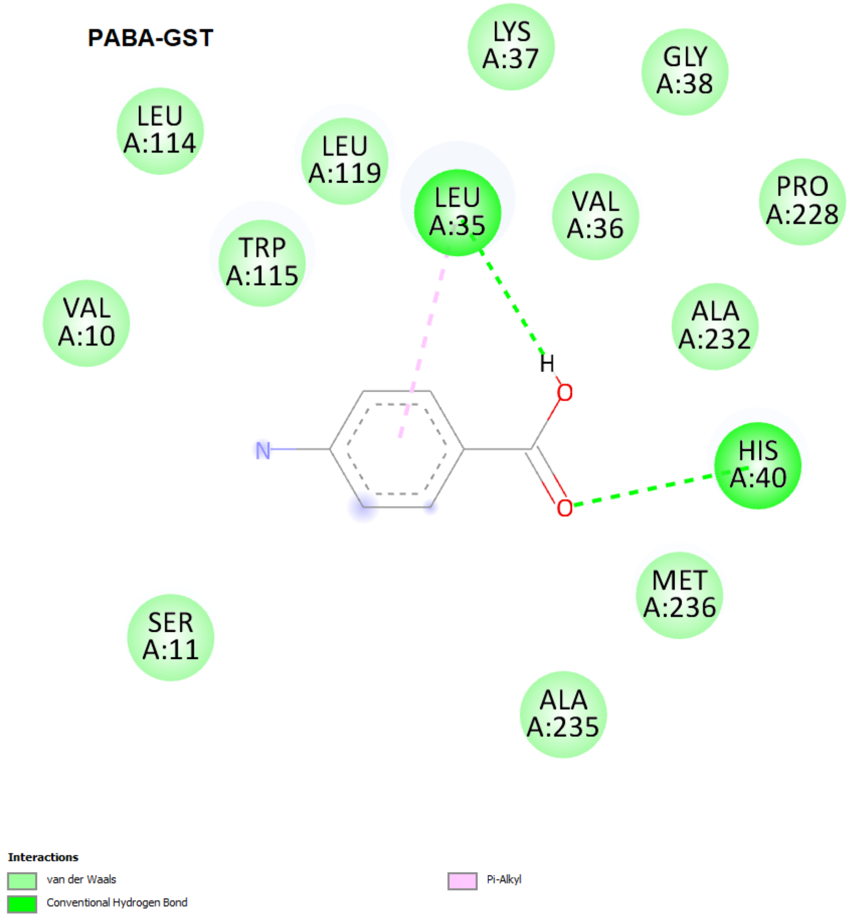


**PABA-NAT2**

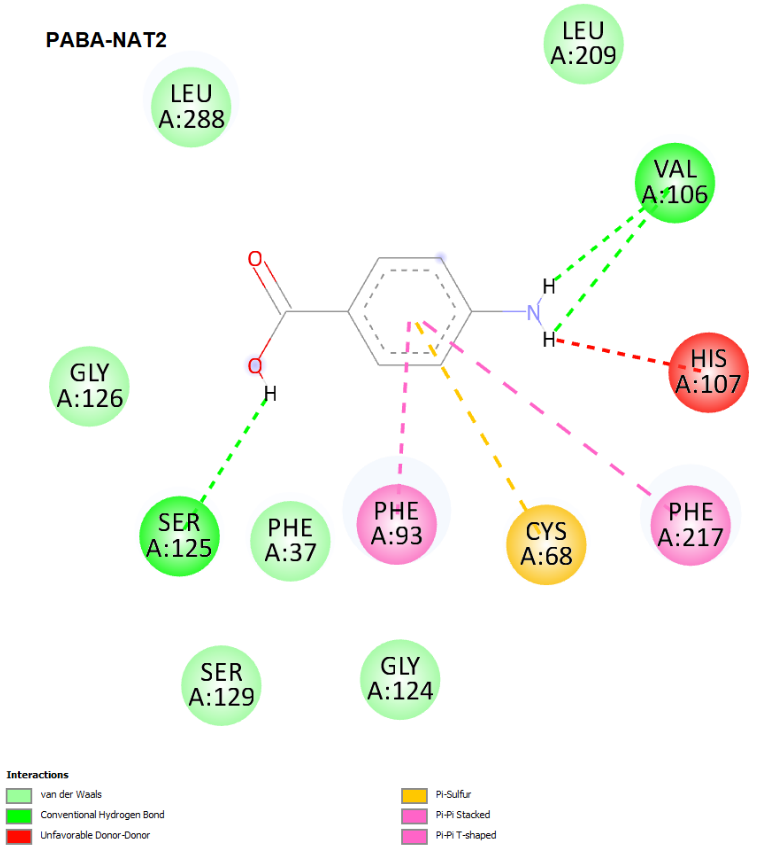

**PBSA-GST**

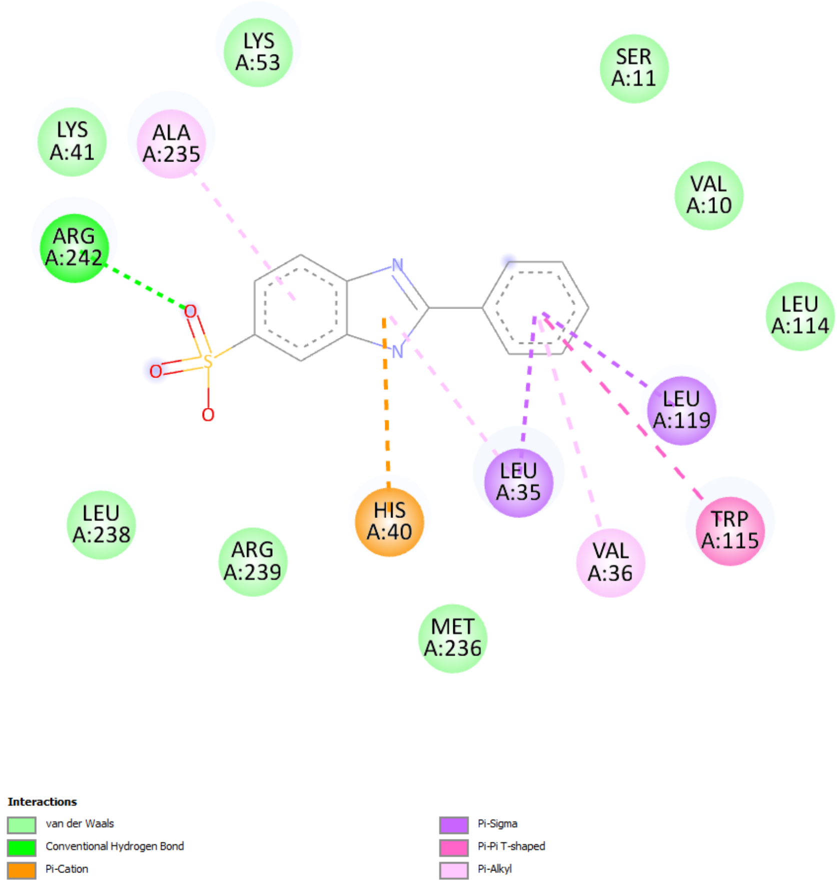


**PBSA-NAT2**

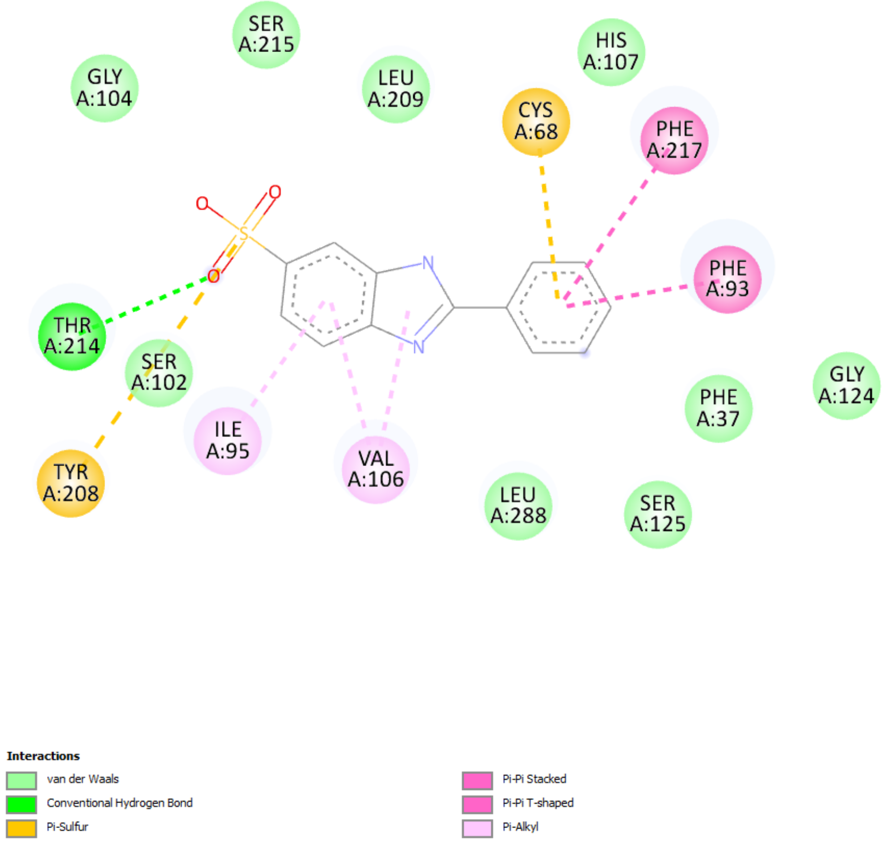

**BMDM-GST**

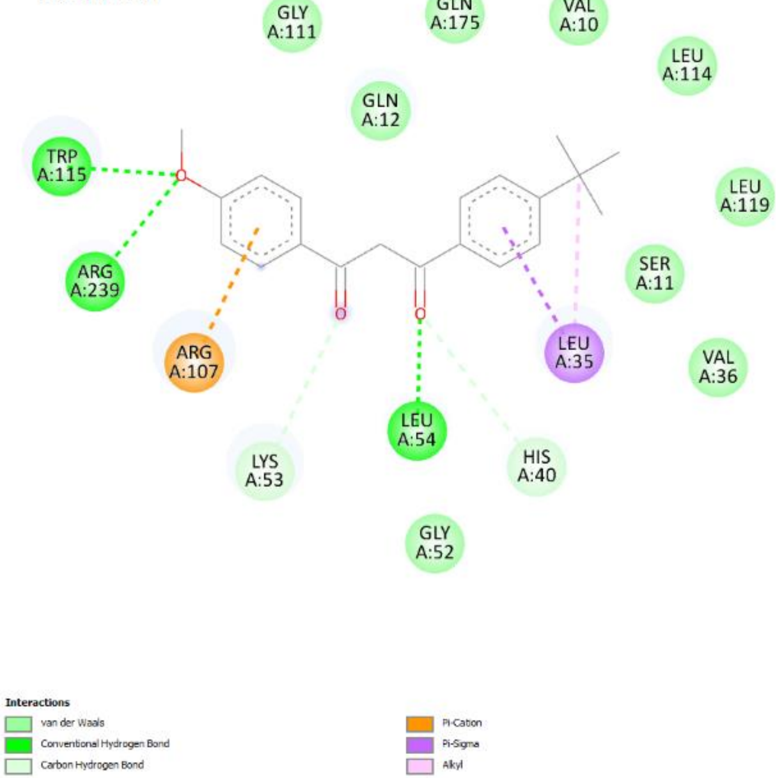


**BMDM-NAT2**

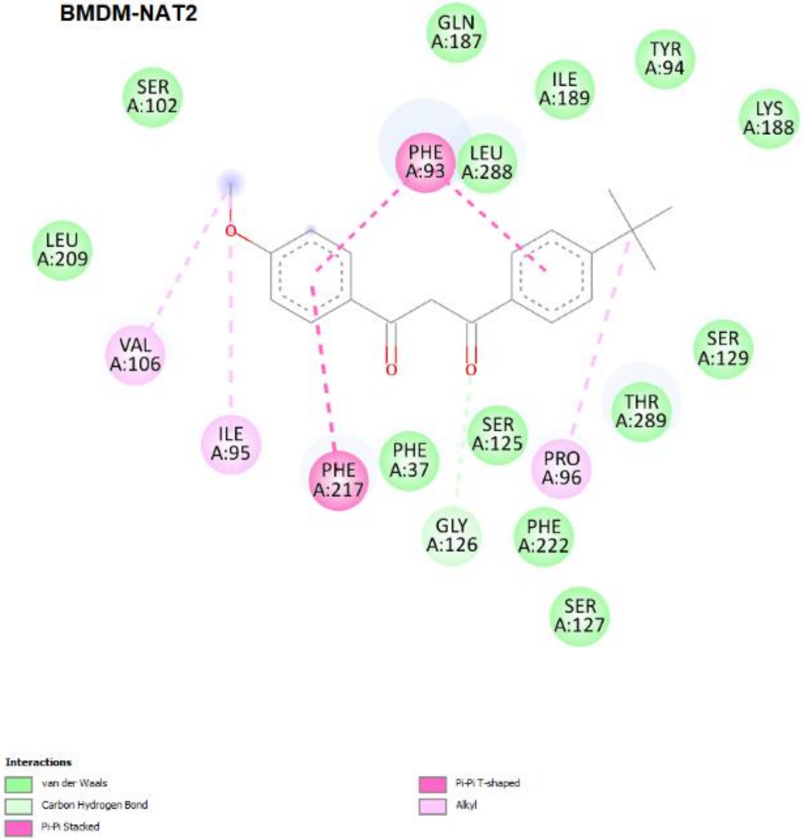

Supplement: Supplementary file 1 [file molecules-29-05836-s001.zip › Supplementary Materials - Figure S1.pdf]
