# Supplementary material for: Organic Sunscreens—Is Their Placenta Permeability the Only Issue Associated with Exposure During Pregnancy? In Silico Studies of Sunscreens’ Placenta Permeability and Interactions with Selected Placental Enzymes
Source: Molecules. 2024 Dec 11;29(24):5836. doi: 10.3390/molecules29245836 (PMC11728689; doi:10.3390/molecules29245836)
Supplement: Supplementary file 1 [file molecules-29-05836-s001.zip › Supplementary Materials - Table S1.pdf]

Table S1. Key molecular descriptors, observed and predicted PL0/PL1 classification of studied compounds

No.	Compound	*AATSC6m*	*VSA_EState5*	*XLOGP3*	*ATSC2d*	*Lipinski*	**Observed PL0/PL1**	ANN1	GDA
1	Oxychlordane	101.9	-0.7859	4.19	-10.74	0	0	0	0
2	DDE	-35.95	0	6.51	-2.84	0	0	0	0
3	Mifepristone	0.05511	7.758	3.77	1.327	0	0	0	0
4	Atazanavir	-4.968	-1.035	5.6	-17.61	0	0	0	0
5	Nonachlor	141.1	-0.8171	6.2	-7.823	0	0	0	0
6	Chlordane	-31.51	-0.4884	6.16	-3.958	0	0	0	0
7	HCB	0	0	5.73	-6	0	0	0	0
8	Flupenthixol	7.228	0	4.44	1.502	1	1	1	1
9	Lopinavir	1.77	-0.04928	5.92	-8.25	0	0	0	0
10	Propranolol	-5.664	0.821	2.98	-1.231	1	1	1	1
11	Disopyramide	7.504	-0.3566	2.58	1.75	1	1	1	1
12	Piperacillin	0.5496	-4.121	0.5	-1	0	1	1	1
13	Heptachlor	-204	-0.4699	4.35	-3.901	0	1	1	1
14	Etidocaine	-0.3723	0.1113	3.69	3.104	1	1	1	1
15	Buprenorphine	-1.808	1.802	4.68	11	1	1	1	1
16	Oxprenolol	-5.273	1.291	2.1	-2.051	1	1	1	1
17	Didanosine	-0.7191	-0.1338	-1.24	1.834	1	1	1	1
18	Norbuprenorphine	-2.361	0.8873	3.47	12.72	1	1	1	1
19	Clindamycin	3.317	0.3497	2.16	-0.6678	1	1	1	1
20	Lidocaine	-4.766	0.0612	2.26	-0.6075	1	1	1	1
21	Clonazepam	-4.697	-0.2804	2.41	-2.191	1	1	1	1
22	Flecainide	-1.839	-1.467	3.78	-4.609	1	1	1	1
23	Nevirapine	-16.37	1.399	1.96	3.844	1	1	1	1
24	Remifentanil	-4.814	-0.7757	1.88	4.025	1	1	1	1
25	Ethabutol	17.94	0	-0.44	-3.567	1	1	1	1
26	Nifedipine	-0.01232	-2.327	2.2	11.27	1	1	1	1

27	Acebutolol	-5.315	0.1545	1.71	-0.8639	1	1	1	1
28	Clonidine	-34.13	0.7222	1.98	-0.7297	1	1	1	1
29	Ticarcillin	-1.705	-5.079	0.81	-2.685	1	1	1	1
30	Lamivudine	-1.089	0.8168	-0.93	-0.2574	1	1	1	1
31	Chlorpyrifos	24.49	0.05213	4.96	8.188	1	1	1	1
32	Indomethacin	-4.235	-0.5832	4.27	4.546	1	1	1	1
33	Metronidazole	-14.01	0.4558	-0.02	2.286	1	1	1	1
34	Diazinon	-11.49	1.366	3.81	8.319	1	1	1	1
35	Metoprolol	-6.96	0.7822	1.88	-4.796	1	1	1	1
36	Abacavir	-8.549	1.184	0.87	0.6805	1	1	1	1
37	Procainamide	7.556	-0.0443	0.88	-3.501	1	1	1	1
38	Zidovudine	-4.225	0	0.05	-2.178	1	1	1	1
39	Diazepam	6.613	-0.02824	2.99	-0.1405	1	1	1	1
40	Stavudine	-7.417	0	-0.81	-1.765	1	1	1	1
41	Valproic acid	-10.39	-0.7373	2.75	-1.414	1	1	1	1
42	Indinavir	-2.377	-0.7814	2.92	-14.18	0	0	0	0
43	Duloxetine	-0.7782	0.9607	4.32	2.859	1	0	1	1
44	17-hydroxyprogesterone caproate	-0.2637	1.661	5.65	5.404	0	0	0	0
45	Nelfinavir	0.5329	1.272	5.67	-12.33	0	0	0	0
46	Bupivacaine	-1.738	0.1696	3.41	0.7147	1	1	1	1
47	Cefoperazone	2.364	-4.261	-0.74	-3.228	0	1	1	1
48	Naloxone	1.551	0.5282	2.09	10.01	1	1	1	1
49	Isoniazid	6.937	4.581	-0.7	-2.055	1	1	1	1
50	Midazolam	3.363	0.5761	2.48	2.985	1	1	1	1
51	Phthalimide	5.669	-0.6007	1.15	-0.9258	1	1	1	1
52	Chloroquine	5.134	0	4.63	1.5	1	1	1	1
53	Sotalol	-2.087	0	0.24	-4.809	1	1	1	1
54	Dicloran	-4.74	0	2.8	-4.5	1	1	1	1

BMDM	Butyl methoxydibenzoylmethane	11.31	0.3421	4.76	-10.51	1	1	1
BP3	Benzophenone-3	-7.119	0.2464	3.79	-1.136	1	1	1
DHHB	Diethylamino hydroxybenzoyl hexyl benzoate	-0.7649	-0.9687	6.5	10.41	0	0	0
PABA	4-aminobenzoic acid	15.77	-0.9306	0.83	-4.408	1	1	1
EHDP	Ethylhexyl dimethyl 4-aminobenzoate	1.36	0.2687	5.03	-2.694	1	1	1
Et-PABA	Ethyl- 4- aminobenzoate	-5.37	-0.3079	1.86	-0.6238	1	1	1
PBSA	Phenylbenzimidazole sulphonic acid	-3.935	0.6556	1.99	-3.411	1	1	1
MBC	4-methylbenzylidene camphor	0.01254	0.82	4.51	-5.193	1	1	1
EHMC	Ethylhexyl 4-methoxycinnamate	-0.6727	1	5.3	-2.685	1	1	1
IMC	Isoamyl p-methoxycinnamate	1.545	1.043	4.47	-4.047	1	1	1
OCR	Octocrylene	-4.192	-0.2075	7.14	5.528	0	0	0
ET	Ethylhexyl triazone	0.8399	0.6742	14.54	2.81	0	0	0
OS	2-Ethylhexyl salicylate	-5.049	-0.04916	5.71	-0.3725	1	1	1
HMS	Homosalate	9.24	0.1199	5.05	-5.946	1	1	1
DOBT	Diethylhexyl butamido triazone	-0.566	0.5241	11.82	-3.087	0	0	0
BP4	Sulisobenzone	-7.235	-1.256	2.18	0.9587	1	1	1
